# KCa3.1 channels regulate the tumor infiltration of functionally competent NK cells in head and neck cancer

**DOI:** 10.1038/s41598-025-20101-x

**Published:** 2025-10-17

**Authors:** Abdulaziz O. Alshwimi, Ameet A. Chimote, Marat V. Khodoun, Layne Weatherford, Benjamin H. Hinrichs, Heike Wulff, Stephen N. Waggoner, Trisha M. Wise-Draper, Laura Conforti

**Affiliations:** 1 Division of Nephrology, Department of Internal Medicine, University of Cincinnati, Cincinnati, OH USA; 2 Division of Rheumatology, Department of Internal Medicine, University of Cincinnati, Cincinnati, OH USA; 3 Division of Immunobiology, Cincinnati Children’s Hospital Medical Center, Cincinnati, OH USA; 4 Division of Hematology Oncology, Department of Internal Medicine, University of Cincinnati, Cincinnati, OH USA; 5 Department of Pathology and Laboratory Medicine, University of Cincinnati, Cincinnati, OH USA; 6 Department of Pharmacology, University of California Davis, Davis, CA USA; 7 Department of Pediatrics, University of Cincinnati, Cincinnati, OH USA; 8 Center for Autoimmune Genomics and Etiology, Division of Human Genetics, Cincinnati Children’s Hospital Medical Center, Cincinnati, OH USA

**Keywords:** Tumour immunology, Innate immune cells

## Abstract

CD8^+^ T cells and natural killer (NK) cells are the primary defenses against tumor cells. The tumor microenvironment impairs their antitumor capabilities, including the ability to infiltrate the tumor. Our laboratory has shown that tumors suppress T cells by inhibiting KCa3.1 K^+^ channel activity that controls cytokine release, cytotoxicity, and chemotaxis. However, little is known about the role of K^+^ channels in the anti-tumor activity of NK cells. Here, we investigated KCa3.1 channels’ role in human NK cell function in head and neck squamous cell carcinoma (HNSCC). Selective blockade of KCa3.1 using TRAM-34 inhibited chemotaxis of NK cells from both healthy donors and patients with HNSCC, while KCa3.1pharmacological activation increased chemotaxis and cytotoxicity. SKA-31, a selective KCa3.1 activator, enhanced antitumor immune responses in a humanized HNSCC mouse model generated by implanting Cal27 cells and healthy donor peripheral blood mononuclear cells into immunodeficient mice. SKA-31 treatment resulted in a 7-fold increase in total NK cells and a 23-fold increase in functionally active (granzyme B-positive) NK cells in these tumors. These data highlight the critical role of KCa3.1 channels in antitumor immunity and open the possibility of targeting KCa3.1 to develop new immunotherapies for HNSCC.

**Supplementary Information:**

The online version contains supplementary material available at 10.1038/s41598-025-20101-x.

## Introduction

Natural killer (NK) cells are cytotoxic cells that kill target cells via perforin and granzymes released from cytolytic granules or via death receptor pathways. They function as the first-line defense against infections and cancer. NK cells identify and eliminate stressed cells, including cancer cells, in part because of the loss or downregulation of ligands, including major histocompatibility complex I (MHC-I), for inhibitory NK cell receptors on the surface of putative target cells. The absence of MHC-I in target cells can trigger NK cell effector responses, leading to a form of cell killing termed missing self^1^. Because NK cell killing requires physical proximity to the target cells, NK cells must migrate to and infiltrate tumors^2^. The trafficking of cytotoxic T cells and NK cells into the tumor microenvironment (TME) is a key step in effective cancer immune surveillance.

In this study, we focus on NK cells in head and neck cancer, specifically head and neck squamous cell carcinoma (HNSCC), the most common type. HNSCC comprises malignancies that develop in the mucosal membranes lining various areas of the head and neck region, including the pharynx, larynx, and oral cavity^3^. HNSCC is the 6th most common cancer worldwide, with an estimated 58,450 new cases and 12,230 projected deaths in the United States alone by 2024^4^.

NK cells play an essential role in tumor surveillance and control in HNSCC, and their presence is associated with a favorable prognosis^5^. Higher NK cell infiltration has been reported in HNSCC than in other tumor types^6^. Additionally, patients with higher levels of tumor-infiltrating NK cells, particularly the highly cytotoxic CD56^dim^ NK cells, tend to have better outcomes^7^. Nevertheless, NK cells typically exhibit reduced cytotoxic activity and CD16 expression in patients with HNSCC and mouse models of this disease. NK cells from patients with HNSCC also produce less interferon-γ (IFNγ) than cells from healthy donors (HDs)^8^. Consequently, emerging therapies aimed at enhancing the activity of NK cells as an effective strategy against HNSCC are being developed^9,10^. However, a better understanding of the mechanisms regulating NK cell function in cancer is essential for the development of new strategies to improve antitumor immunity.

Ion channels are known regulators of immune cell function; however, although they have been well characterized in T lymphocytes, information on ion channels in NK cells remains limited^11,12^. The importance of ion channels in immune cells lies in their ability to control the Ca^2+^ influx necessary for multiple functions, including cytokine production, differentiation, proliferation, and motility^13,14^. Ca^2+^ influx is the result of the concerted functions of K^+^ and Ca^2+^ channels. K^+^ channels maintain the electrochemical gradient necessary for Ca^2+^ influx through the Ca^2+^channels^15^. The main K^+^ channels expressed in NK cells are the voltage-dependent K^+^ channel Kv1.3, Ca^2+^-activated potassium channel KCa3.1, and two-pore domain channel TWIK-related acid-sensitive K^+^ channel 2 (TASK2)^16–18^. In T cells, both Kv1.3 and KCa3.1 have been shown to contribute to antitumor immunity with KCa3.1, the focus of our investigation, regulating chemotaxis, cytokine release, and responses to immunosuppressive adenosine and programmed death ligand 1 (PDL1)^12,19–21^. Overall, KCa3.1 acts as a positive regulator of T cell function. Blocking KCa3.1 suppresses chemotaxis and cytokine release in T cells^19^. However, little is known about the K^+^ channels in NK cells. TASK2 has been implicated in NK cell proliferation and cytolytic function^18^. Likewise, Kv1.3 is involved in the degranulation and cytotoxicity of NK cells^16^. Inhibition of these K^+^ channels suppresses cytotoxicity^16–18^. Interestingly, KCa3.1 appears to function differently in NK cells and T cells. KCa3.1 blockade in NK cells increases cytotoxicity, proliferation, and degranulation, suggesting KCa3.1 may act as a negative regulator of NK cell antitumor function^16,17^. In this study, we investigated the role of KCa3.1 channels in human NK cell chemotaxis and effector functions. We provide evidence of the positive regulatory roles of KCa3.1 channels in NK cell chemotaxis in HNSCC and suggest that KCa3.1 activators may offer therapeutic benefits by enhancing the ability of NK cells to infiltrate the HNSCC TME.

## Materials and methods

### Human subjects

Peripheral blood was obtained from 12 treatment-naïve patients with HNSCC aged 38–83 years at the University of Cincinnati Medical Center (Table 1 for patient demographics). The patient inclusion criteria comprised a positive HNSCC diagnosis that was confirmed by tissue biopsy and no radiation or chemotherapy treatment prior to phlebotomy. Research Electronic Data Capture (REDCap) tools hosted by the University of Cincinnati were used to maintain patient information. Peripheral blood was also collected from 29 HDs. Twenty-eight of these 29 HD samples were discarded from the Hoxworth Blood Center (University of Cincinnati), whereas one HD sample was obtained by phlebotomy from a healthy volunteer. Age and demographic information of the samples obtained from the Hoxworth Blood Center were not available. Informed consent was obtained from all individuals and patients participating in the study. The study and the informed consent documents received approval from the University of Cincinnati Institutional Review Board (IRB numbers. 2013 − 1950, 2014–4755 and 2013–2516). All experiments on human subjects were performed in accordance with relevant guidelines and regulations.

**Table 1:** Demographic information of enrolled HNSCC patients. Patients who fitted the inclusion criteria were enrolled in the study (*n* = 12). Eastern cooperative oncology group (ECOG) performance status describes how the disease affects the patient’s care for themselves and their daily performance. The extent and size of tumors define the stages from T1 to T4. The regional lymph node involvement is categorized from N1 to N3 depending on the number of lymph nodes and their location. N0 = absence of cancer in the regional lymph nodes. For smoking status evaluation, pack years are calculated by multiplying the number of packs of cigarettes smoked in a day by the number of years the patient has smoked.

Age (at the time of sample collection)	Years
Range	38 to 83
Mean	62

Variable	Number (%)
Gender	
Male	10 (83)
Female	2 (17)
T1	2 (17)
T2	4 (33)
T3	
T4	5 (42)
Unknown	1 (8)
Nodal status	
N0	6 (50)
N1	1 (11.1)
N2	4 (33)
N3	
Unknown	1 (8)
ECOG performance status	
0	3 (25)
1	3 (25)
2	
Unknown	6 (50)
Smoking	
No (< 10 pack years)	3 (25)
Yes (≥ 10 pack years)	9 (75)
Alcohol	
No	7 (58)
Yes (≥ 5 drinks/week)	5 (42)
p16 status	
Positive	2 (17)
Negative	5 (42)
Unknown	5 (42)

### Reagents and chemicals

Human serum, 1-EBIO, and sodium hydroxide (NaOH) were purchased from Millipore. HEPES, RPMI 1640, fetal bovine serum, penicillin, streptomycin, L-glutamine, minimum essential amino acids, sodium pyruvate, and phosphate-buffered saline (PBS) were obtained from Gibco (Thermo Fisher). DMEM and rat tail collagen I were purchased from Corning Inc., NS309 and ShK-Dap22 were purchased from Tocris Bioscience, and CXCL10 (cat#266-IP/CF) was obtained from R&D Systems. TRAM-34 and SKA-31 were provided by Dr. H. Wulff (Department of Pharmacology, University of California, Davis, CA, USA). Stock solutions of TRAM-34, 1-EBIO, and NS309 were prepared in dimethyl sulfoxide (DMSO) and used at 0.05% dilution^22^. Stock solutions of CXCL10, IL-2 (Peprotech, cat#200-02), IL-15 (BioLegend, cat#570304), IL-12 (Peprotech, cat#200-02), and IL-18 (BioLegend, cat#570304) were prepared in sterile PBS containing 0.1% bovine serum albumin.

### Cell lines and cell culture

Cal27 cells were obtained from the American Type Culture Collection (ATCC; cat#CRL-2095), UMSCC cells were procured from Millipore Sigma (cat# SCC071), and HN5 cells were a generous gift from Dr. Nira Ben-Jonathan, Department of Cancer Biology, University of Cincinnati. Cal27 and HN5 cell lines were cultured in DMEM supplemented with 10% FBS, 1% penicillin-streptomycin, 1% minimum non-essential amino acids, 1% sodium pyruvate, and 6 mM L-glutamine. UMSCC cells were cultured in DMEM supplemented with 10% FBS, 1% penicillin-streptomycin, high glucose, L-glutamine, and sodium pyruvate. Cells were maintained in a humidified 5% CO_2_ incubator at 37 °C.

### Cell isolation and activation

Peripheral blood mononuclear cells (PBMCs) were isolated from whole blood samples using a Ficoll-Paque (Cytiva) density gradient. NK cells were isolated from PBMCs by negative selection using the EasySep Human NK Cell Enrichment Kit (STEMCELL Technologies, Cat. #19055). Primary NK cells were maintained in RPMI 1640 medium containing L-glutamine supplemented with 10% fetal bovine serum, penicillin, 1% streptomycin, and 100 IU/ml IL-2. Cells were activated using 100 IU/ml IL-2 and 10 ng/ml IL-15 for 48 h or 10 ng/ml IL-12 and 100 ng/ml IL-18 for 24 h. All cells were maintained in a 5% CO_2_ incubator at 37 °C.

### Chemotaxis

A three-dimensional (3D) chemotaxis assay utilizing a µ-Slide chamber (ibidi GmBH) was performed as previously described^19^. Briefly, 1 × 10^6^ activated NK cells were incorporated into type I rat tail collagen (Corning, cat. #354236) to form a gel that was added to the central compartment of the µ-slide chamber. A chemokine gradient was created by adding CXCL10 (8 µg/ml) to the reservoir to the left of the central compartment^19^. In some experiments, TRAM-34 (500 nM) and adenosine (ADO) (1 µM) were added to CXCL10 to create a gradient. Non-chemokine conditions (+/− vehicle) were used as controls. Chemotaxis experiments were performed in phenol red-free RPMI medium supplemented with 10% FBS (standard migration medium). For chemotaxis in Ca^2+^-controlled solutions, the following Ringer solution was used: 2 mM Ca^2+^ solution consisting of 155 mM NaCl, 4.5 KCl, 1 mM MgCl_2_, 2 mM CaCl_2_, 10 mM HEPES, and 10 mM glucose (pH 7.4). The Ca^2+^-free solution consisted of 155 mM NaCl, 4.5 mM KCl, 1 mM MgCl_2_, 10 mM HEPES, 10 mM glucose, and 2 mM ethyne glycol tetraacetic acid (EGTA, pH 7.4). Migrating cells were imaged by mounting the µ-slide chamber on the stage of an inverted Zeiss LSM 710 microscope (Carl Zeiss Microscopy GmBH) or a Leica DMi8 inverted microscope (Leica Microsystems) equipped with a 37 °C incubation system. NK cell migration was recorded using time-lapse video microscopy with bright-field images captured every 3 s for Zeiss or 4.8 s for Leica. A total of 1000 images were obtained for each experiment. For cell tracking analysis, we used the “Manual Tracking plugin” for the ImageJ software (National Institutes of Health, 1.52a). The tracked data were analyzed using the Chemotaxis and Migration Tool (ibidi GmBH). Ten to 15 cells were tracked for each condition. We quantified the following chemotactic parameters: center of mass (COM; the average point of single cells traveled by the end of the experiment), Euclidean distance (the linear distance between the starting and ending points of a cell), accumulated distance (the total distance traveled by the cell by the end of the experiment), forward migration index (FMI, which represents the efficiency of forward migration of the cells towards the chemokine gradient), directness (the measure of the straightness of the cell trajectory), and velocity^19^. We considered the chemotaxis effect to be positive if the cells migrated along the chemokine gradient (y-axis), meaning that the COM along the y-axis (Y-COM) was significantly greater than that along the x-axis (X-COM), and the FMIy (FMI along the y-axis) was significantly greater than that along the x-axis (FMIx).

### Electrophysiology

KCa3.1 and Kv1.3 currents were recorded with a whole-cell voltage clamp configuration using an AxoPatch 200 B amplifier (Molecular Devices), as previously described^14,20^. The external solution contained 145 mM NaCl, 5 mM KCl, 1 mM MgCl_2_, 2.5 mM CaCl_2_, 5.5 mM glucose (D-(+)-dextrose), and 10 mM HEPES (pH 7.4) (all reagents were purchased from Millipore Sigma). The pipettes were pulled from borosilicate glass (World Precision Instruments, TW150F-4) by using a P-97 puller (Sutter Instruments). The pipette solution contained 145 mM K-aspartate, 10 mM EGTA, 8.5 mM CaCl_2_, 2 mM MgCl_2_, and 10 mM HEPES at pH 7.2 (1µM free Ca^2+^) (all reagents from MilliporeSigma). Currents were obtained by 200-ms ramp depolarization from − 120 mV to + 50 mV every 15 s from a holding potential of −70 mV. The KCa3.1 conductance was measured from the slope of a linear equation between − 100 mV and − 85 mV, and the ratio of the slope of the linear current (I) to the slope of the ramp voltage (V) was determined by leak current subtraction. Kv1.3 currents were measured using the same ramp recordings at + 50 mV after eliminating extrapolated KCa3.1^23^. KCa3.1 slope conductance was measured between − 100 mV and − 60 mV. Data were corrected for a liquid junction potential of − 10 mV (https://pmc.ncbi.nlm.nih.gov/articles/PMC1456048/). The digitized signals were stored and analyzed using pClamp 9 software (Axon Instruments).

### Animals

Immune-deficient NOD/LtSz-SCID IL-2RG−/− (NSG) mice were obtained from the Comprehensive Mouse and Cancer Core (Division of Experimental Hematology and Cancer Biology) at Cincinnati Children’s Hospital Medical Center. Six-week-old mice of both sexes were used for the experiments and were flank injected with 100 µl of Cal27 cell suspension (16 × 10^6^ cells in 200 µl solution containing 50% PBS and 50% Matrigel). When the tumors were palpable (approximately 21 days post-injection), each mouse received 10 × 10^6^ PBMCs in 0.5 ml i.v. plus 10 × 10^6^ cells in 0.5 ml s.c. on the perimeter of the tumor but not inside. One group of mice (*n* = 4 mice) was treated with 30 mg/kg of the KCa3.1 activator SKA-31 (5 µl/g of mouse weight) i.p. daily for 13 days, starting on the day of PBMC injection (*n* = 4 mice). Another group of mice (*n* = 4) was treated with vehicle. Fourteen days later, the mice were sacrificed, and the tumors were harvested. A stock solution of SKA-31 was freshly prepared by dissolving 12 mg SKA-31 in 2 ml of peanut oil, warmed, and dissolved at 70–80 °C on a stirrer in a 50 ml aluminum capped bottle until completely dissolved^24^. Injections were administered immediately thereafter. The mice were euthanized via CO_2_ inhalation and subsequently sacrificed for tumor excision at 11–12 weeks of age. At the time of sacrifice, the weights of the mice in both the SKA-31 and control groups ranged from 27 to 35 g, with no significant difference observed between the groups (*p* = 0.80). In these groups, tumor volumes at the time of sacrifice ranged from 21 to 81 mm^3^. The study protocol received approval from the Institutional Animal Care and Use Committee (IACUC) of Cincinnati Children’s Hospital Medical Center (IACUC protocol numbers: 2020-0073, 2018-0038, 2024-0064, 21-10-29-01), and all efforts were made to minimize animal suffering. All mouse experiments were performed in accordance with relevant guidelines and regulations. The study adhered to the ARRIVE guidelines 2.0 for reporting animal research, specifically following the recommendations for detailed descriptions of animal characteristics, experimental procedures, and statistical methods.

### Immunohistochemistry

For NK cell infiltration, HNSCC mouse tumors were harvested. The tumors were then formalin-fixed paraffin-embedded (FFPE) and sectioned at 5 μm thickness at The Pathology Research Core at Cincinnati Children’s Hospital Medical Center (CCHMC). Tumor slides were deparaffinized and stained with anti-human CD161 antibodies (marker of NK cells; Abcam cat# ab197979) and granzyme B (Thermo Fisher Scientific, cat#MA1-35461) in a Ventana BenchMark ULTRA automated immunohistochemistry (IHC) slide staining system (Roche AG), as described previously^19^. To further confirm the presence of NK cells, slides were stained with another NK cell marker, CD56 (CellMarque; cat#156 M-88), while tumor regions were confirmed by staining with pancytokeratin (Roche AG; cat#760–2595). Primary antibodies were detected indirectly using an ultraView Universal 3,3’-diaminobenzidine (DAB) Detection Kit (Ventana Medical Systems) containing a horseradish peroxide multimer and 3,3′-diaminobenzidine tetrahydrochloride (DAB) chromogen. Slides were stained with hematoxylin (Roche AG, cat#760–2021). Images were obtained at 40X magnification using a Leica DMi8 inverted microscope with Leica Application Suite X software (Leica Microsystems). At least ten fields were imaged per slide. For image analysis, the tumor regions and tumor periphery were assessed using pan-keratin staining for each slide. NK cell infiltration within the intratumoral region and the tumor periphery was digitally quantitated by drawing a region of interest (ROI) around the tumor (or periphery) region using ImageJ software with a manual drawing tool. Infiltrated NK cells (brown signal) in each ROI were quantified digitally, normalized to the area measured in the ROI using ImageJ, and expressed as cells counted per square millimeter. We then quantified the NK cells that also expressed a granzyme B signal (purple signal) in each ROI in the same way using ImageJ and characterized them as the percentage of granzyme B-positive NK cells.

### Reverse transcription quantitative polymerase chain reaction (RT-qPCR)

Total RNA was isolated from resting and activated HD CD8^+^ T cells from different HNSCC cell lines (HN5, Cal27, and UMSCC-47) and from three HNSCC patient tumor biopsies using the E.Z.N.A. Total RNA Isolation Kit (Omega Bio-tek), according to the manufacturer’s instructions. RNA (600 ng) was used to synthesize complementary DNA (cDNA) using the Maxima First Strand cDNA Synthesis Kit for RT-qPCR (Thermo Fisher Scientific) according to the manufacturer’s instructions. Predesigned TaqMan Gene Expression Assay primers (Applied Biosystems, Thermo Fisher Scientific) were used to detect the expression of *KCNN4* (assay ID: Hs01069779_m1) and *18S rRNA* (assay ID: Hs99999901_s1). RT-qPCR was performed in a 96-well plate by adding 30 ng of cDNA, 1× TaqMan Gene Expression Master Mix (Applied Biosystems, Thermo Fisher), and 1 µl of TaqMan Gene Expression Assay primers. For technical replicates, all samples were run in quadruplicates. RT-qPCR was performed using an Applied Biosystems StepOne Real-time PCR system (Applied Biosystems). * 18S rRNA* was used as an internal control. C_T_ values were measured using StepOne software, version 2.1 (Applied Biosystems, Thermo Fisher). ΔC_T_ values for *KCNN4* were calculated by normalizing the measured C_T_ values of *KCNN4* to the measured C_T_ values of * 18S rRNA*, and ΔΔC_T_ values were calculated as previously described^19^. Relative quantity (RQ) values, representing the fold-change in *KCNN4* gene expression compared to activated HD CD8^+^ T cells, were calculated as the 2^−ΔΔCT^ values.

### Proliferation

To assess the effect of the KCa3.1 activator NS309 on HNSCC cell proliferation, proliferation assays were conducted on two HNSCC cell lines (Cal27 and UMSCC-47) using the CellTiter 96 Aqueous One solution kit (MTS) (Promega, cat#G3582) according to the manufacturer’s instructions. Cells (2 × 10^3^ cells/well) were incubated for 24, 48, or 72 h in the presence of 1, 5, or 10 µM NS309, whereas cells incubated with DMSO (vehicle) were used as controls. For technical replicates, samples were run in quintuplicates. The samples were read according to the manufacturer’s instructions, and the optical density (OD) value measured in the blank sample was subtracted from the OD values measured for the samples. The data are presented as the percentage of proliferating cells in NS309 treated samples normalized to untreated (vehicle-treated) samples.

### Multiplex cytokine release

To investigate the cytokine and cytotoxicity marker release profiles of NK cells, we used a fluorescence bead-based LEGENDplex™ Human CD8/NK Panel (BioLegend; cat#741187) comprising the following detectable analytes: IL-17 A, IL-2, IL-4, IL-10, IL-6, tumor necrosis factor α(TNF-α), soluble Fas (sFas), soluble Fas-ligand (sFasL), IFN-γ, granzyme A, granzyme B, perforin, and granulysin. The assay was performed according to the manufacturer’s instructions. Briefly, 1 × 10^6^ HD NK cells were activated with either IL-2 and IL-15 for 48 h or IL-12 and IL-18 for 24 h, in the presence or absence of 500 nM TRAM-34 or 1 µM NS309, respectively. We also activated HNSCC NK cells with IL-2 and IL-15 for 48 h. Twenty-five microliters of cell culture supernatants from activated HD and HNSCC NK cells were used for the assay, which was performed in duplicate in a 96 well plate according to the manufacturer’s instructions. The samples were acquired on a Cytek^®^ Aurora flow cytometer equipped with a yellow-green laser and an automated 96-well plate reader using the SpectroFlo^®^ software and using instrument settings provided by the manufacturer, and the resultant FCS (flow cytometry standard) files were analyzed using the LEGENDplex™ Data Analysis Software as per the manufacturer’s protocol. The data for the analytes in HD NK cells were reported as the fold-change in the mean fluorescence intensities of the analytes in the presence of TRAM-34 or NS309 treated NK cells relative to the control cells.

### Cytotoxicity assay

To evaluate the effect of KCa3.1 channel modulation on the killing potential of NK cells, in vitro cytotoxicity assays were conducted using Cal27 spheroids. Cal27 cells (1,000 cells per well) were seeded in a 96-well U-bottomed Nunclon Sphera plate (ThermoFisher Scientific, Cat #174925) in complete Cal27 medium, which was formulated with phenol red-free FluoroBrite DMEM (ThermoFisher Scientific, Cat #A1896701) and FBS. After centrifugation at 120 × g for 5 min, the plate was incubated at 37 °C with 5% CO_2_ in a humidified incubator. Spheroid formation was visually confirmed after 48 h. NK cells were isolated from healthy donor PBMCs and activated with IL-2 and IL-15 for 48 h, either with or without 500 nM TRAM-34 or 1 µM NS309. The activated NK cells were labeled with Cell Tracker Deep Red dye (ThermoFisher, Cat#C34565) following the manufacturer’s protocol and resuspended in complete medium containing phenol red-free RPMI. The labeled NK cells were co-cultured with Cal27 spheroids at an effector-to-target ratio of 10:1 for 3 h. The positive controls included spheroids (not co-cultured with NK cells) treated with 1% Triton-X-100 (MilliporeSigma) for 1 h, while the negative controls were spheroids without NK cells. To assess cell killing, a solution with CellEvent Caspase-3/7 Green ReadyProbes Reagent (indicator of apoptotic cells, ThermoFisher, Cat #R37111) in phenol red-free medium was prepared according to the manufacturer’s instructions. This solution was added to the co-cultures for 30 min before imaging.

Imaging was performed using a Zeiss LSM 710 microscope (Carl Zeiss Microscopy GmBH) equipped with an incubation system at 37 °C. Z-stack images (0.5 μm thickness) were acquired using a 10x objective lens with the pinhole set at 1 airy unit. The Zeiss Zen Image browser (Zeiss GmbH) was used for image acquisition, employing the “Multi Track” option to prevent channel cross-talk. Image analysis was conducted using ImageJ (Fiji) software (NIH, Bethesda, MD, USA). Regions of interest (ROI) were drawn around the spheroid boundary in the focal plane at the center of the spheroid. After adjusting the image threshold and background, the mean fluorescence intensities (mean gray values, adjusted to the ROI area) were measured for the Caspase 3/7 (cell killing) and Cell Tracker Deep Red (NK cell abundance) channels.

### Statistical analysis

Statistical testing was performed using either Student’s *t* test (paired or unpaired) or one- way/repeated-measures analysis of variance (ANOVA) for multiple comparisons. Post hoc testing for ANOVA was performed by conducting multiple pairwise comparisons using Tukey’s multiple comparison test. Normality for each experiment was determined using the Shapiro–Wilk test, and when normality failed, statistical significance was determined using either the Mann-Whitney rank sum test for unpaired comparisons or Wilcoxon signed-rank test for pairwise comparisons. *P* value of less than or equal to 0.05 was defined as statistically significant. All statistical tests were performed using GraphPad Prism 10.2.0 (GraphPad Software LLC, Boston, MA, USA). Appropriate statistical tests and corresponding values are described in individual figure legends.

## Results

### Chemotaxis of activated NK cells is Ca^2+^ dependent

**Fig. 1:**
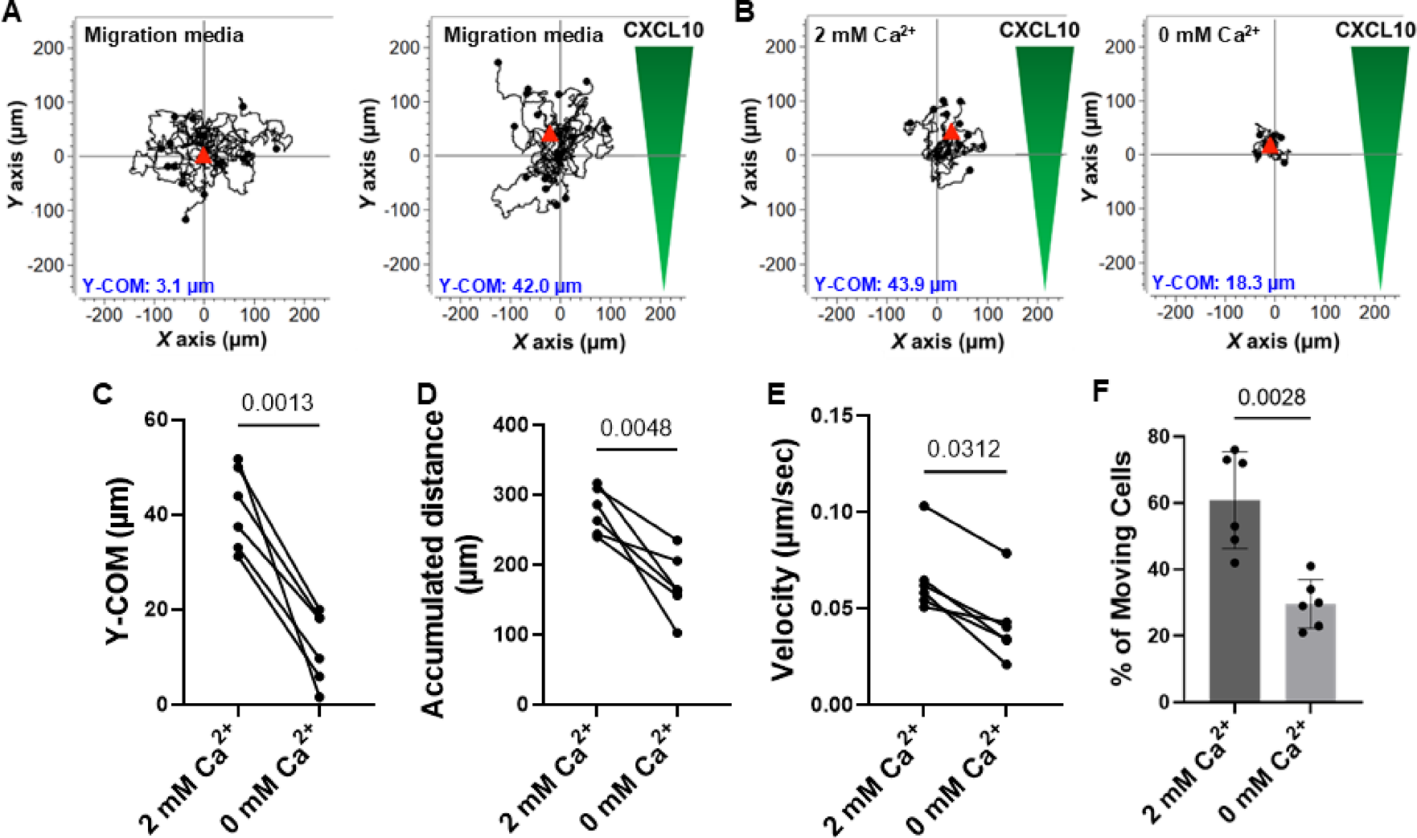
Chemotaxis of activated primary NK cells depends on extracellular Ca^2+^ concentrations. (**A**) Left: Single-cell trajectories of NK cell random migration (without a chemokine gradient) in normal migration media (RPMI). Right: NK cells migrating towards a CXCL10 gradient (green). Cells were maintained in normal migration media (RPMI). (**B**) Single-cell trajectories of NK cells migrating towards a CXCL10 gradient (green) in either 2 mM Ca^2+^ Ringer solution (left) or 0 mM Ca^2+^ Ringer solution (right). Trajectories of 9–14 cells are shown for each experiment, and the starting point of each cell is artificially set to the same origin. The red triangles represent the Y-center of mass (Y-COM), i.e., the Y-coordinate of the average distance traveled by the cells towards the chemokine by the end of the experiment. The value of the Y-COM for each experiment is reported at the bottom of panels A-C. (**C**) Y-COM values, (**D**) Accumulated distance (distance traveled by cells from the start until the end of the experiment), (**E**) velocity, and (**F**) percentage of moving cells in migrating NK cells from 6 HDs along a CXCL10 gradient in either 2 mM or 0 mM Ca^2+^ solutions. Data in (**C**) were analyzed by Wilcoxon signed-rank test, while data in (**D-F**) were analyzed by paired Student’s t-test. Bars represent mean ± SD, and each symbol represents an individual donor.

The ability of NK cells to infiltrate tumors is a critical step in effective cancer control. Understanding the ionic mechanisms that mediate NK cell chemotaxis is essential for designing new immunomodulators that facilitate the ingress of NK cells into tumors. While KCa3.1 channels have been shown to regulate the motility of T lymphocytes by controlling Ca^2+^ oscillations at the uropod of moving cells, the ionic mechanisms governing NK cell motility remain poorly understood^14,20^. We investigated the ability of activated human NK cells to chemotax at different extracellular Ca^2+^ concentrations using a three-dimensional chemotaxis chamber. In this setup, NK cells are mixed with collagen to form a stable 3D collagen matrix for cells to migrate towards a chemoattractant (CXCL10) gradient, mimicking a tumor-like structure^19,25^. All chemotaxis experiments were conducted using primary NK cells isolated from the blood of healthy donors (HD) and activated with IL-2 and IL-15 for 48 h. Activated NK cells exhibited random movements in the absence of a chemokine gradient (Fig. 1A, left). However, they migrated towards high chemokine concentrations in the presence of a CXCL10 gradient in standard migration medium (Fig. 1A, right). To assess dependence on Ca^2+^, we tested NK cell chemotaxis in Ca^2+^-controlled environments using Ringer’s solution with either 2 mM (Fig. 1B, left) or 0 mM Ca^2+^ (Fig. 1B, right). We used Y-COM (y-coordinate of the center of mass, red triangles in Fig. 1) as the unit of measure that defines the chemotactic ability of NK cells throughout most of the manuscript, whereas all other parameters are reported in Tables S1-S5. Y-COM defines the average distance that NK cells travel towards the chemokine gradient. We observed that Y-COM (Fig. 1C) and other parameters associated with active motility towards a chemokine gradient, including accumulated distance (Fig. 1D) and velocity (Fig. 1E), significantly decreased in the absence of Ca^2+^ (0 mM Ca^2+^). We observed a 58% decrease in the percentage of moving cells (Fig. 1F) following Ca^2+^ depletion. Overall, these data showed that Ca²^+^ regulates NK cell motility. Because KCa3.1 channels control both Ca²^+^ concentrations in human NK cells^17^ and the Ca²^+^-dependent motility of T cells^14^we further investigated their role in NK cell chemotaxis.

### KCa3.1 channels regulate chemotaxis, cytokine release and cytotoxicity in activated primary NK cells from healthy donors

**Fig. 2:**
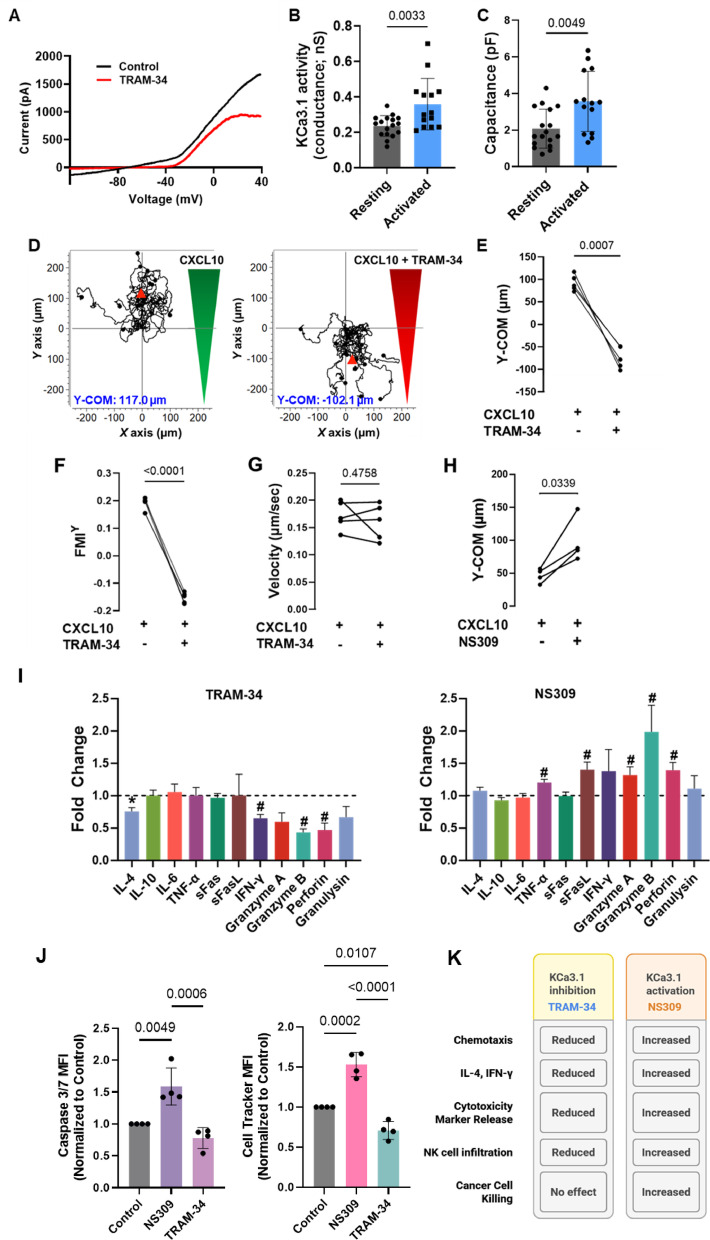
KCa3.1 channels regulate NK cell chemotaxis, cytokine release and cytotoxicity. (**A**) Representative KCa3.1 current traces in activated NK cells from a healthy individual before and after exposure to the selective KCa3.1 blocker TRAM-34 (200 nM). KCa3.1 activity was elicited by depolarizing ramp-pulses from − 120 mV to + 50 mV (holding potential: −70 mV) in whole-cell configuration with 1 µM free Ca^2+^ in the pipette. (**B**) Average KCa3.1 activity (reported as conductance) and (**C**) average cell capacitance recorded in 17 resting NK cells and 14 activated NK cells from 4 HDs. (**D**) Single-cell trajectories of a representative experiment of NK cells migrating towards a CXCL10 gradient (green; left) or a combination gradient of CXCL10 and 500 nM TRAM-34 (red; right). Trajectories of 15 cells are shown for each experiment, and the starting point of each cell is artificially set to the same origin. The red triangles represent Y-COM. (**E**) Y-COM, (**F**) Forward migration index-Y (FMI^Y^), i.e., the directed active cell movement towards the Y-axis, and (**G**) Velocity of migrating cells measured in activated NK cells migrating along a CXCL10 gradient, or a combination gradient of CXCL10 and TRAM-34 (*n* = 5 HDs). (**H**) Y-COM values calculated for activated NK cells migrating towards a CXCL10 gradient with or without 1µM NS309 (a KCa3.1 activator) preincubation in healthy donors (*n* = 4 donors). (**I**) Multiplex cytokine release assay showing fold change in the abundance of individual proteins in activated primary NK cells that were treated with either 500 nM TRAM-34 (left) or 1 µM NS309 (right). Vehicle treated cells were used as controls. The abundance of the individual analyte represented in the bars is shown as relative to that in controls (dotted line). Data were measured in *n* = 5 HDs (the same donors were used for both treatments). **(J)** Bar graphs showing mean fluorescence intensities (MFI) of caspase 3/7 fluorescence, indicating cell death (left) and Cell Tracker Dye, representing the abundance of NK cells (right) in Cal27 spheroids co-cultured with activated HD NK cells that were treated with either 1 µM NS309 or 500 nM TRAM-34. Vehicle-treated cells served as controls. The MFIs for the NS309 and TRAM-34 groups were normalized to the MFI of the control group. Data were collected from four HDs. **(K)** Effects of KCa3.1 inhibition and activation on NK cell function (summary of results in Panels D-J). Data for **(B**,** C)** were analyzed by unpaired Student’s t-test, for **(E-G)** by paired Student’s t-test. Data in **(H**,** I)** were analyzed by Wilcoxon signed-rank test. Bars represent mean ± SD, and each symbol represents an individual donor. In **(I)** significance denoted by (*) for *P* ≤ 0.05 and (#) for *P* ≤ 0.001. Data in **(J)** were analyzed with one-way ANOVA (*P* = 0.006 for caspase MFI, and *P* < 0.001 for Cell Tracker MFI), posthoc testing (significant P values shown in graphs) was performed by Tukey’s test.

Electrophysiological experiments showed that activated NK cells express a high number of functionally active KCa3.1 channels (Fig. 2A-B), as reported by others^16,17^. We measured KCa3.1 currents in whole-cell patch-clamp experiments in NK cells activated with IL-2 and IL-15, using a high Ca^2+^ concentration in the pipette solution to trigger KCa3.1 channel opening, as previously described^20,21^. Currents were elicited with depolarizing ramp-pulses from − 120 mV to + 50 mV (holding potential: −70 mV) (Fig. 2A). The selective KCa3.1 blocker TRAM-34 effectively inhibited KCa3.1 currents (Fig. 2A)^22^. From the current recordings, we calculated the conductance of KCa3.1, a measure of KCa3.1 activity (see Materials and Methods). KCa3.1 conductance was calculated to be between − 100 mV and − 80 mV, where the contamination due to Kv1.3 channels (which open at depolarized voltages) is minimal^20,26^. We observed a significant increase in KCa3.1 conductance in activated NK cells compared to resting NK cells (Fig. 2B), along with an increase in cell capacitance (Fig. 2C). Cell capacitance serves as a marker of activation, reflecting the increase in cell size that occurs in NK cells upon activation^17^. Additionally, we observed an increase in functional Kv1.3 channels in activated NK cells (Fig. S1) as previously described^16^.

To establish the role of KCa3.1 in NK cell chemotaxis, we conducted 3D chemotaxis experiments in HD NK cells. Blockade of KCa3.1 with TRAM-34 significantly suppressed NK cell chemotaxis (Fig. 2D-F). We compared chemotaxis in a chemokine gradient (Fig. 2D, left) with that of a chemokine and KCa3.1 blocker (TRAM-34) (Fig. 2D, right). KCa3.1 blockade decreased NK cell chemotaxis, as indicated by a 180% reduction in Y-COM (Fig. 2E) and 183% reduction in the y coordinate for the forward migration index (FMI^Y^), a chemotaxis parameter that reflects the directed active NK cell movement towards the Y-axis (Fig. 2F). However, we observed no effect of KCa3.1 blockade on velocity (Fig. 2G) or other parameters (Table S2). To further confirm the vital role of KCa3.1 channels in NK cell chemotaxis, we utilized the pharmacological KCa3.1 activator NS309^27,28^. NS309 enhances the mechanical coupling of Ca^2+^ to calmodulin, a Ca^2+^ sensor bound to the intracellular C-terminal domain of KCa3.1, thereby facilitating channel opening^29^. The cells were pretreated with NS309 prior to chemotaxis experiments. NS309 increased NK cell chemotaxis, as indicated by the increase in Y-COM in the NS309-treated group (Fig. 2H). This finding was confirmed using another pharmacological KCa3.1 activator, 1-EBIO (Fig. S2)^19^.

To assess the effect of KCa3.1 on other effector functions of NK cells, we conducted a multiplex cytokine release assay. This bead-based assay allowed us to simultaneously measure multiple cytokine and cytotoxicity markers: interlukin-4 (IL-4), IL-10, IL-6, TNFα, sFas, sFasL, IFNγ, granzyme A, granzyme B, perforin, and granulysin. We quantified the release of these proteins in the presence or absence of either TRAM-34 or NS309 in IL-2 and IL-15- activated NK cells from five donors (same donors for both TRAM-34 and NS309, Fig. 2I). These experiments showed significant inhibition of IL-4, IFNg, and all the cytotoxicity markers except granulysin when the KCa3.1 blocker TRAM-34 was added (compared to the normalized untreated controls, represented by a dotted line, Fig. 2I, left). Conversely, KCa3.1 activation by NS309 significantly increased TNF-a, sFasL, and all the cytotoxicity markers except granulysin (Fig. 2I, right). Comparable results were obtained for NK cells activated with IL-12 and IL-18 (Fig. S3). Further cytotoxicity studies revealed that NS309 enhanced NK cell infiltration into the HNSCC spheroids and cancer cell killing (Fig. 2J, Fig. S4). Interestingly, TRAM-34, even at a concentration sufficient to inhibit cytokine release (Fig. 2I), did not affect cytotoxicity (Fig. 2J, Fig. S4), despite reducing NK cell infiltration into the HNSCC spheroids (Fig. 2J, Fig. S4). Thus, KCa3.1 channels play a role in chemotaxis and cytokine production in human NK cells, and their activation boosts NK cell killing capacity (Fig. 2K). We then proceeded to determine the functional state and role of K^+^ channels in NK cells from HNSCC patients.

### Reduced KC3.1 channel activity in NK cells from HNSCC patients correlates with impaired chemotactic abilities

**Fig. 3:**
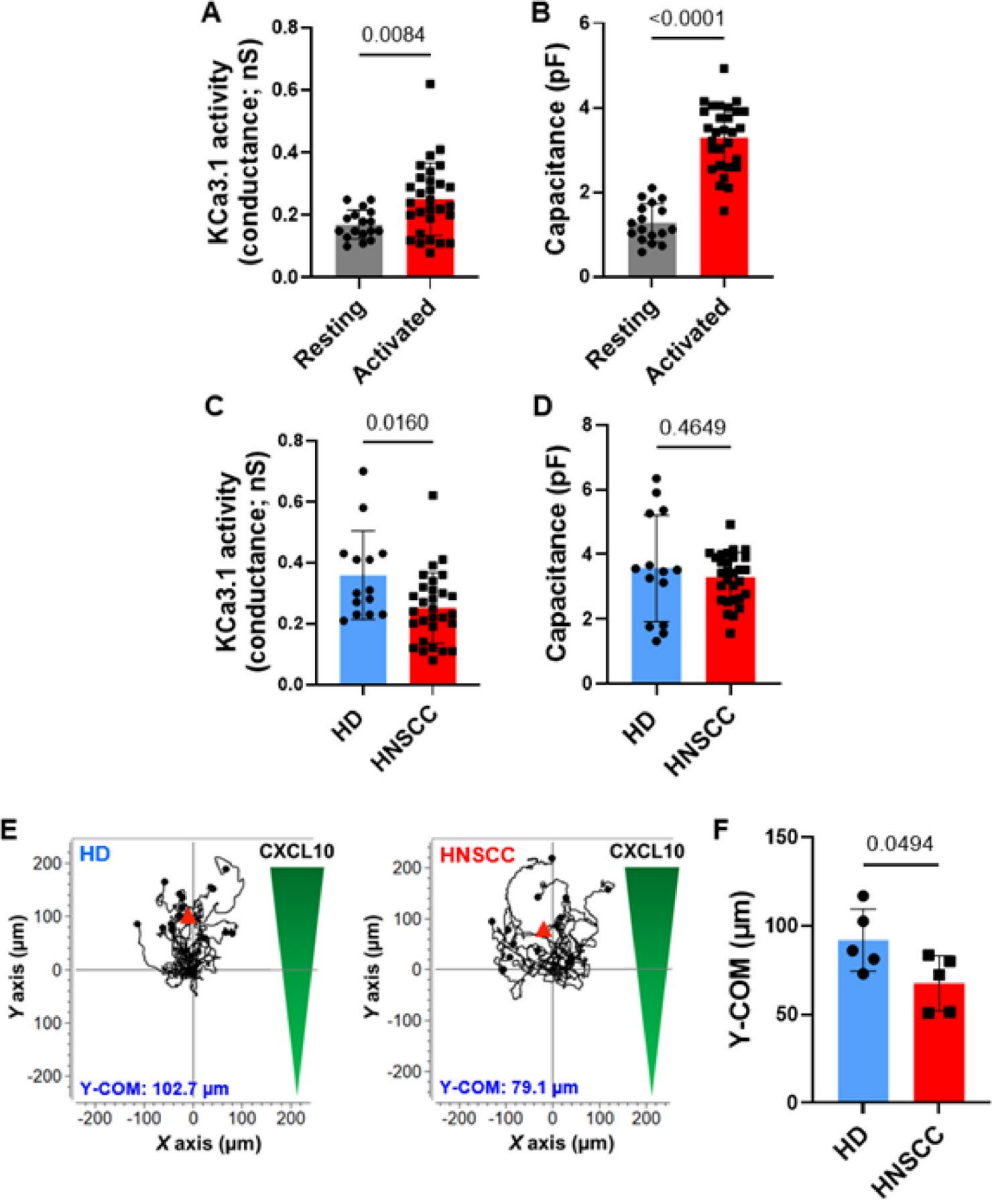
HNSCC patients have reduced levels of functional KCa3.1 channels in NK cells compared to healthy donors. (**A**) Average KCa3.1 activity (reported as conductance) and (**B**) average cell capacitance recorded in resting and activated NK cells from HNSCC patients (17 resting and 29 activated cells from 8 HNSCC patients). (**C**,** D**) Comparison of KCa3.1 activity and cell capacitance between **(C)** healthy donors (HD) and **(D)** HNSCC patients in activated NK cells (14 cells from 4 HDs and 29 cells from 8 HNSCC patients). The data for HDs are the same data reported in Fig. 2B. (**E**) Single-cell trajectories of activated NK cells from a representative healthy donor (left) and HNSCC patient (right) migrating towards a chemokine (CXCL10) gradient. Trajectories of 15 cells are shown for each experiment, and the starting point of each cell is artificially set to the same origin. The red triangles represent Y-COM. (**F**) Average Y-COM of activated NK cells migrating towards a CXCL10 gradient in HD (*n* = 5) and HNSCC (*n* = 5) individuals. Data were analyzed by either unpaired Student’s t-test **(A**,** B**,** D**,** F)** or Mann-Whitney test **(C).** Bars represent mean ± SD. Each symbol represents either an individual cell **(A-D)**, or an individual donor for **(F)**.

We investigated the functionality of KCa3.1 channels in NK cells freshly isolated from the blood of treatment-naïve patients with HNSCC. The characteristics of the HNSCC patients included in this study are shown in Table 1. We assessed KCa3.1 channel activity in HNSCC NK cells using whole-cell patch-clamp experiments, using the same activation method and electrophysiological protocol described above for HD NK cells. Similar to HD NK cells, the activity of KCa3.1 channels (Fig. 3A) and cell capacitance (Fig. 3B) in HNSCC patient NK cells increased upon activation. Additionally, there was an increase in the Kv1.3 functional channels (Fig. S5A). When comparing KCa3.1 channel activity between activated HNSCC and HD NK cells, we observed a significant decrease in KCa3.1 (Fig. 3C) and Kv1.3 (Fig. S5B) activity in HNSCC cells compared to HD cells, whereas no differences were detected in cell capacitance (Fig. 3D). These findings suggest that although NK cells from HNSCC patients activate similarly to those from HDs, they do not achieve the same levels of functional K^+^ channels. Consequently, circulating NK cells in HNSCC patients may be less capable of performing effector functions than HD NK cells.

We observed a reduced chemotaxis capacity of HNSCC NK cells compared to that of HD NK cells. Consistent with the differences in KCa3.1 channels (Fig. 3E, F), HNSCC NK cells exhibited a 27% reduction in Y-COM relative to HD NK cells. We did not observe any changes in other chemotactic parameters (Table S3). No significant differences in cytokine release were observed between HNSCC NK cells and HD NKs, except for an increase in granzyme B and granulysin levels in HNSCC NK cells (Fig. S6). However, given the high variability in these two cohorts, drawing firm conclusions based on the limited sample size was not possible. Nevertheless, the functional consequences of reduced KCa3.1 in HNSCC circulating NK cells warrant further investigation.

### KCa3.1 channels regulate chemotaxis of HNSCC patient-derived NK cells in a tumor-like environment

**Fig. 4:**
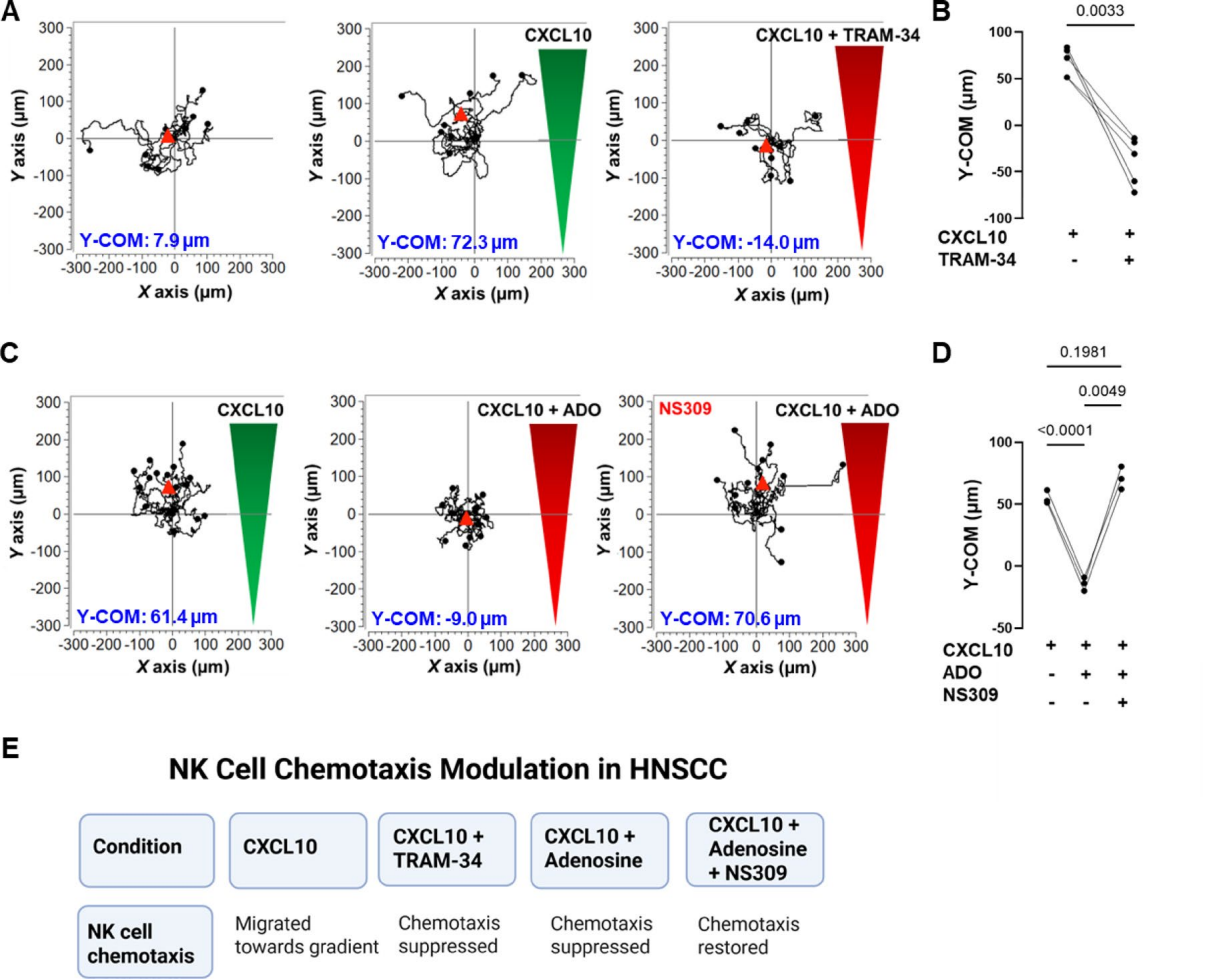
KCa3.1 channels regulate HNSCC NK cell chemotaxis and the response to adenosine. **(A)** Single-cell trajectories of a representative experiment of activated NK cells migrating in the absence of chemokine (left), towards a CXCL10 gradient (middle, green), or a combination gradient of CXCL10 and TRAM-34 (right, red). Trajectories of 15 cells are shown for each experiment, and the starting point of each cell is artificially set to the same origin. The red triangles represent the Y-center of mass (Y-COM). **(B)** Y-COM values calculated for HNSCC NK cells migrating along a CXCL10 gradient, or a combination gradient of CXCL10 and TRAM-34 (*n* = 5 HNSCC patients). **(C)** Single-cell trajectories of a representative experiment of activated NK cells migrating towards either a CXCL10 gradient (left, green) or a mixed gradient of CXCL10 and 10 µM adenosine (ADO) (red, middle), or a mixed gradient of CXCL10 and ADO (red, right). The right panel shows cells migrating in a CXCL10/ADO gradient after pretreatment with 1µM NS309. **(D)** Y-COM values calculated for HNSCC NK cells migrating along a CXCL10 gradient, or a combination gradient of CXCL10 with ADO with or without preincubation with NS309 (*n* = 3 HNSCC patients). **(E)** Schematic of effects of KCa3.1 modulation on NK cell chemotaxis in HNSCC. Data were analyzed by paired Student’s t-test for **(B)**, and repeated measures one-way analysis of variance (*P* = 0.004) with Grisser-Greenhouse correction for **(D)**. Post-hoc testing to assess pairwise comparisons in **(D)** was done by Tukey’s multiple comparison test.

We investigated whether the chemotaxis of circulating NK cells from HNSCC patients depends on KCa3.1. We performed 3D chemotaxis experiments using CXCL10 gradient in the presence or absence of TRAM-34. Similar to HD NK cells, HNSCC NK cells migrated randomly in the absence of chemokines (Fig. 4A, left). Conversely, they migrated toward the chemokine gradient in the presence of CXCL10 (Fig. 4A, middle). However, when TRAM-34 was added to the chemokine gradient, NK cell chemotaxis was suppressed (Fig. 4A, right panel; Table S4). Quantification of Y-COM in primary NK cells from five separate HNSCC patients confirmed the significant inhibition of chemotaxis upon treatment with a KCa3.1 blocker (Fig. 4B). We observed comparable inhibition of TRAM-34 in HNSCC NK cells (Y-COM = 155 ± 13%) and HD NK cells (Y-COM = 180 ± 9%) (Figs. 2E and 4B, *P* = 0.1569). All chemotaxis parameters of HNSCC NK cells in the presence or absence of TRAM-34 are shown in Table S4.

The dependence of HNSCC NK cell chemotaxis on KCa3.1 could have a high impact on the ability of NK cells to infiltrate the TME. The HNSCC TME is rich in immunosuppressive nucleoside adenosine, which is generated by cancer, stroma, and regulatory T cells (Tregs)^30,31^. We have previously shown that adenosine inhibits KCa3.1 channels and chemotaxis in HNSCC CD8^+^ T cells^20^. Our chemotaxis experiments revealed that HNSCC NK cells were also inhibited by adenosine (Fig. 4C, middle), and this effect was prevented by KCa3.1 activation using NS309 (Fig. 4C, right). Figure 4C-D illustrates the response of NK cells to adenosine and the restoration of NK cell chemotaxis by KCa3.1 activation in three separate HNSCC patients. All chemotaxis parameters before and after adenosine treatment of HNSCC NK cells with or without preincubation with NS309 are reported in Table S5. These data suggest that a similar mechanism may operate in HNSCC tumors, and that KCa3.1 activators could potentially enhance NK cell antitumor activity. To test this hypothesis, we conducted in vivo experiments using a humanized HNSCC mouse model. A schematic summarizing these findings is presented in Fig. 4E.

### Activation of KCa3.1 channels potentiates NK cell tumor infiltration and antitumor function in vivo

**Fig. 5:**
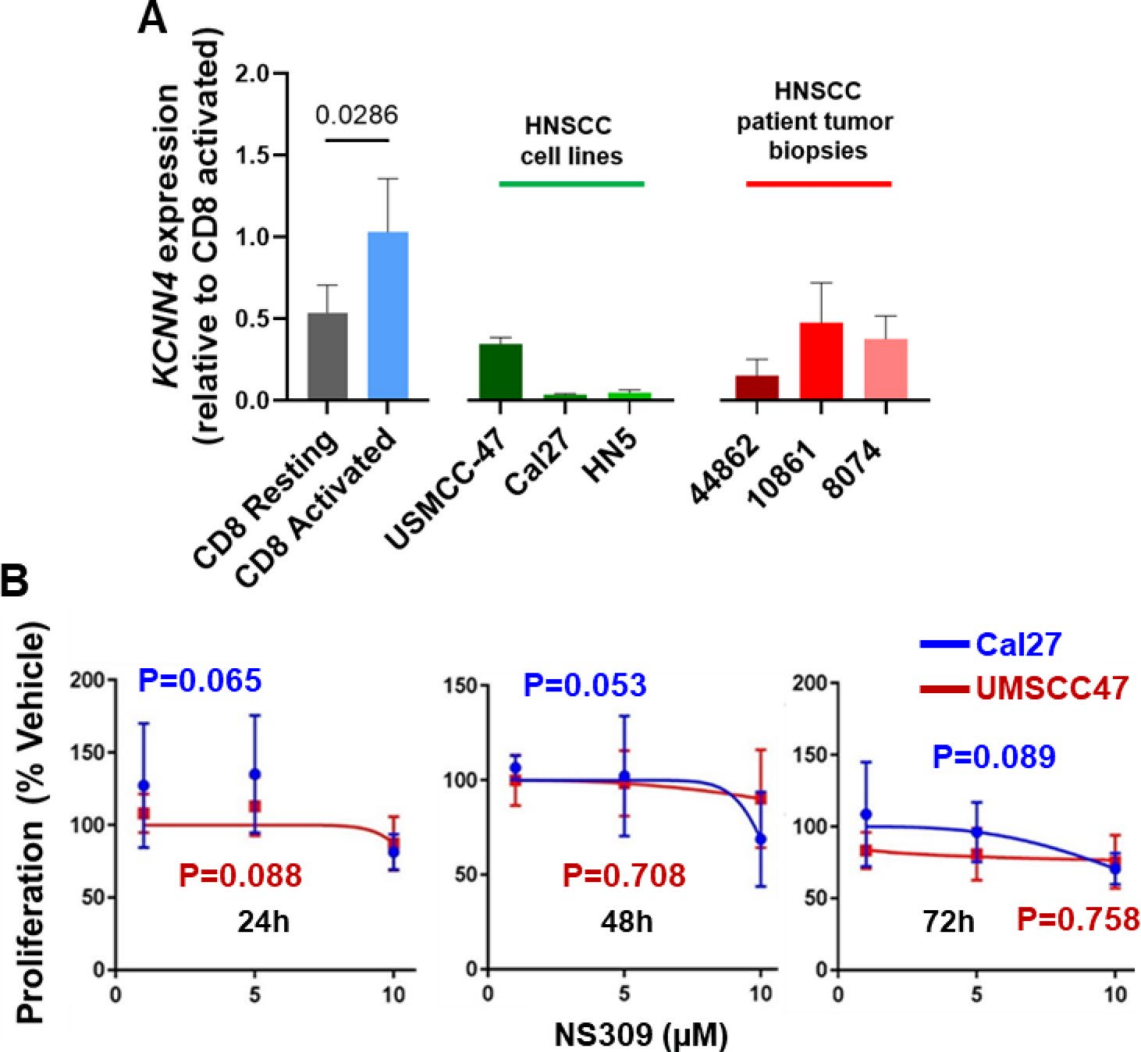
KCa3.1 does not regulate activation in HNSCC cancer cell proliferation. (**A**) *KCNN4* (the gene encoding KCa3.1) mRNA levels were quantified reverse transcription quantitative polymerase chain reaction (RT-qPCR). Data shown are fold change in KCNN4 expression in resting and activated primary CD8^+^ T cells (left), HNSCC cell lines (middle), and HNSCC patient tumor biopsies (right). The data are normalized to the KCNN4 expression levels in activated CD8^+^ T cells. Each sample was run in quadruplicate. 18 S rRNA was used as the housekeeping gene. The bars represent mean ± SD, and each symbol corresponds to an individual experiment. (**B**) Effect of activation of KCa3.1 channels by NS309 on the proliferation of HNSCC cell over various time points up to 72 h. Proliferation was measured at 24, 48 and 72 h in Cal27 (blue line) and UMSCC (red line) HNSCC cell lines treated with 1, 5 and 10 mM NS309 using a colorimetric cell proliferation assay. Data are shown as percent change in proliferation of the HNSCC cells for the different NS309 concentrations as compared to untreated controls. Data in **(A)** were analyzed by unpaired student’s t-test by comparing the KCNN4 abundance in the individual HNSCC cell lines and tumor biopsies and resting CD8^+^ T cells to activated CD8^+^ T cells. Data in **(B)** were analyzed by one-way analysis of variance (ANOVA), and the P-values for the analysis of variance are shown in blue for the Cal27 cells and in red for the UMSCC cells. (P = 0.065 and P = 0.088 for 24h Cal27 and UMSCC respectively, P = 0.052 and P = 0.708 for 48 h Cal27 and UMSCC respectively, and P = 0.089 and P = 0.7577 for 72 h Cal27 and UMSCC respectively).

Before investigating whether KCa3.1 activation could boost NK cell antitumor activity in HNSCC, we first needed to rule out any off-target effects on cancer cells. The literature contains conflicting evidence regarding the role of KCa3.1 in cancer cells. Glioblastoma and breast cancer cells express KCa3.1, which facilitates their proliferation^32,33^. However, other studies have challenged the tumorigenic potential of the KCa3.1^34,35^. Our data indicated that HNSCC cells expressed low levels of KCa3.1 and that KCa3.1 activation did not increase their proliferation (Fig. 5). To assess KCNN4 (the gene encoding KCa3.1) expression, we conducted reverse transcription quantitative polymerase chain reaction (RT-qPCR) experiments on three HNSCC patient tumor biopsies and three immortalized human HNSCC cell lines, including HPV-negative HN5 and Cal27 cell lines and the HPV-positive UMSCC-47 cell line^36^. We used resting and activated CD8^+^ T cells for comparison as they express a known number of KCa3.1/cell, about 20 and 500 KCa3.1/cell, respectively^26^. We observed a small to null abundance of KCa3.1 in HNSCC lines and patient tumors, which was comparable to resting T cells (Fig. 5A). Furthermore, functional studies revealed that KCa3.1 activation with NS309 did not alter the proliferation of HNSCC cells, as determined by a colorimetric proliferation assay with two different HNSCC cell lines, Cal27 and UMSCC-47 (Fig. 5B). These findings are encouraging to investigate the potential use of KCa3.1 activators in HNSCC in vivo to enhance NK cell antitumor responses.

**Fig. 6:**
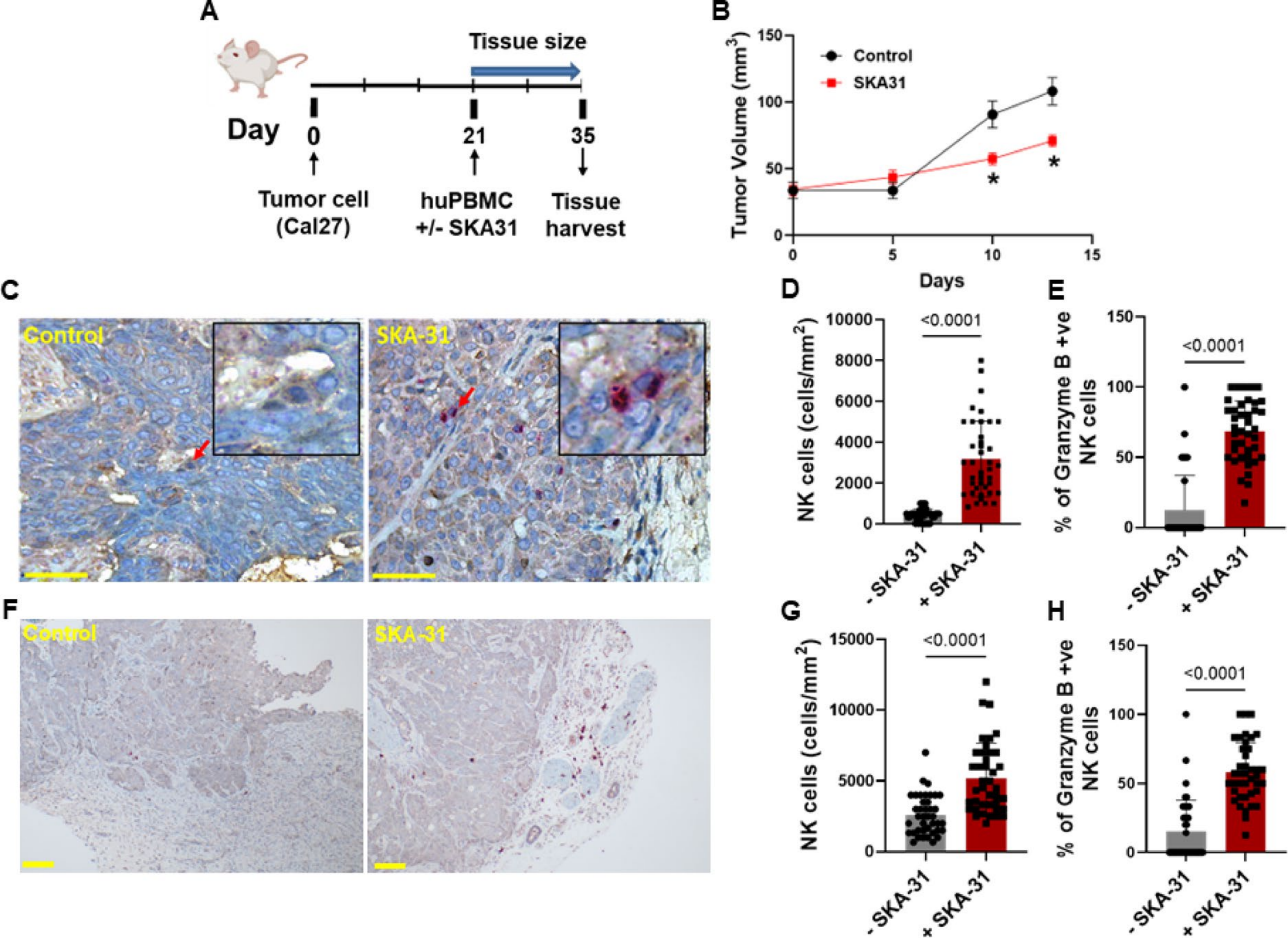
KCa3.1 activation reduces tumor burden and increases the intratumoral abundance of cytotoxic NK cells in a HNSCC mouse model. (**A**) Schematic of HNSCC humanized mouse model generation of NSG mice injected with Cal27 cells (a human HNSCC cell line) on day 0, followed by engraftment of human healthy donor PBMCs +/- SKA-31 (30 mg/kg) injections on day 21. The tumor tissue size was measured between days 21–35 and on day 35, the tumors were harvested for immunohistochemical (IHC) staining. (**B**) Effect of SKA-31 treatment on tumor burden (size measured in mm^3^) (4 mice/group; * *P* < 0.05 by unpaired t-test). Day 0 corresponds to day 21 in panel A, i.e., the day PBMCs were injected. (**C**) Immunohistochemistry of CD161 (dark brown signal; NK cells) and granzyme B (purple signal) expression in tumors harvested from HNSCC humanized mice. Representative IHC images obtained from a region of interest (ROI) within the tumor area of xenografts, highlighting CD161 and granzyme B staining in the presence or absence of SKA-31 are shown here. Scale bar = 50 μm. (**D**) Intra-tumoral abundance of NK cells in vehicle (-SKA-31) and SKA-31-treated mice, determined as number of cells divided by the area of the ROIs within the intratumoral region of the tissues. (**E**) Granzyme B-positive NK cell density +/- SKA-31 normalized for the area of ROIs. (**F**) Immunohistochemistry of CD161 (dark brown signal; NK cells) and granzyme B (purple signal) expression in tumors harvested from HNSCC humanized mice. Representative IHC images obtained from a region of interest (ROI) from the periphery of tumor biopsies, highlighting CD161 and granzyme B staining in the presence or absence of SKA-31 are shown here. **(G)** Abundance of NK cells in the tumor periphery, in vehicle (-SKA-31) and SKA-31-treated mice, determined as number of cells divided by the area of the ROIs within the periphery region of the tissues. (**H**) Granzyme B-positive NK cells, in the periphery of the tumor tissues, in SKA-31-treated and untreated (vehicle, -SKA-31) mice. For each biopsy, 10 fields (ROIs) were imaged, and the quantification was analyzed using an unpaired Student’s t-test. Bars represent mean ± SD, and each symbol represents an individual measurement from the analyzed fields from 4 mice/group.

To study the effect of KCa3.1 activation on NK cell antitumor functions in vivo, we developed a preclinical HNSCC mouse model by implanting Cal27 cells in the flank of immunodeficient NOD/SCID/IL-2Rgamma^−/−^ (NSG) mice (Fig. 6A). When the tumors became palpable (around day 21), we injected peripheral blood mononuclear cells (PBMCs) from a healthy donor intravenously and around the perimeter of the tumor. One group of mice was treated with the KCa3.1 activator naphtho[1,2-d] thiazol-2-ylamine (SKA-31), whereas the other group was treated with vehicle (control group). We used SKA-31 because it a well-established KCa3.1 activator with superior pharmacokinetic properties to NS309 or EBIO^24,28^ Although NS309 is the most potent KCa3.1 activator (EC₅₀ ~70–100 nM), its short half-life (~ 20 min) and off-target effects (hERG and Cav inhibition) limit its in vivo use. EBIO has a much lower potency (EC₅₀ ~28 µM) and is mainly suitable for in vitro studies. SKA-31, NS309, and EBIO share similar selectivity (~ 10-fold for KCa3.1 over KCa2), but SKA-31 has a half-life of 12 h^37^. Overall, SKA-31 offers a balance of potency and selectivity for KCa3.1.

After 35 days, the tumors were excised. During this period, we measured tumor volume using an external caliper. SKA-31 treatment significantly reduced the tumor burden (Fig. 6B). Immunohistochemistry (IHC) analysis of formalin-fixed paraffin-embedded (FFPE) xenograft sections stained for the NK cell marker CD161 and granzyme B (Fig. 6C) revealed a 7-fold increase in CD161^+^ NK cells infiltrating the tumor (identified by positive pancytokeratin staining) compared with that in control mice (Fig. 6C-D). Notably, a higher percentage of NK cells infiltrating the tumor in the SKA-31 treated group were granzyme B-positive (Fig. 6E). Additionally, we observed an increased accumulation of CD161^+^GranzymeB^+^ NK cells at the tumor periphery in the SKA-31 treatment group (Fig. 6F-H). These findings provide the first evidence supporting the potential benefits of KCa3.1 activators in HNSCC treatment.

## Discussion

Failure of immune surveillance and response to immunotherapy in cancer is often attributed to limited infiltration of functionally competent cytotoxic T and NK cells into the tumor. The prevention of physical interactions between these effector cells and tumor cells blunts the effective cytotoxic activity^38^. Hence, favorable cancer prognosis, not only for HNSCC but also for other solid tumors, is associated with a high number of functionally active NK cells infiltrating the tumor^39,40^. In this study, we demonstrated that KCa3.1 channels positively regulate chemotaxis and release of cytokines and cytotoxicity in human NK cells. Furthermore, in vivo experiments in an HNSCC mouse model showed that the KCa3.1 pharmacological activator SKA-31 effectively increased the infiltration of functionally competent (granzyme B-positive) NK cells in tumors and reduced tumor burden. These findings support the use of KCa3.1 activators in HNSCC.

In this study, we demonstrated that KCa3.1 regulates NK cell chemotaxis. Previous reports have shown that KCa3.1 channels control chemotaxis in human CD3^+^ T cells^19,25^. Chemotaxis is a Ca^2+^-dependent process, and here we confirmed that NK cells also rely on extracellular Ca^2+^ for chemotaxis^41^. In T cells, KCa3.1 and TRPM7 channels are necessary for cell motility localized at the uropod, where Ca^2+^ oscillations regulate cell movement, possibly through actomyosin contractility^14,41^. Although it remains uncertain whether this mechanism, downstream of KCa3.1, applies to NK cells, we provide evidence that the role of KCa3.1 in NK cell chemotaxis is equivalent to that observed in CD3^+^ T cells. Specifically, the KCa3.1 blocker TRAM-34 inhibits NK cell chemotaxis, whereas KCa3.1 activators (NS309 and 1-EBIO) enhance it. Notably, TRAM-34 affects Y-COM and FMI but does not affect velocity. In contrast, 0 mM Ca^2+^ suppressed velocity in addition to all other chemotaxis parameters, suggesting additional factors involved in the Ca^2+^-dependent control of NK cell chemotaxis, warranting further investigation.

We conducted these experiments on circulating NK cells activated with IL-2 and IL-15, as resting NK cells express low levels of KCa3.1, which increase upon activation^16,17^. We used this activation method because it matches the method used by others who studied the role of KCa3.1 in NK cells, thus facilitating the comparison of our findings with theirs^16,17^. Contrary to our findings on chemotaxis, Beeton et al. reported no effect of KCa3.1 channels on NK cell migration in a transwell assay^16^. Methodological differences, such as the 2D assay versus our 3D chemotaxis assay in a collagen matrix, and the different cancer type may explain these discrepancies. Overall, our data indicate that chemotaxis in human NK cells is Ca^2+^- and KCa3.1-dependent.

Our results also showed that NK cells rely on KCa3.1 for their chemotaxis. However, we observed that the levels of functional KCa3.1 channels were lower in NK cells from HNSCC patients than in HD NK cells. This reduction in KCa3.1 expression accounted for the decreased chemotactic ability of patient-derived NK cells. Moreover, our study revealed that NK cell chemotaxis in HNSCC was hindered by adenosine and rescued by KCa3.1. This indicates that adenosine, a known immunosuppressive factor present in high concentrations in the TME, could limit NK cell tumor infiltration via KCa3.1 inhibition as shown for CD3^+^ T cells^19^. Additionally, PGE2, another immunosuppressive TME factor and inhibitor of KCa3.1, likely employs a similar mechanism to hinder NK cell tumor infiltration^42^. PGE2 and adenosine have been shown to also reduce NK cell cytotoxicity^43^. Therefore, KCa3.1 activators could serve as a promising strategy to counteract multiple immunosuppressive elements within the TME, enhance NK cell chemotaxis, and promote tumor infiltration in HNSCC.

Cytokine release and cytotoxicity are Ca^2+^-dependent processes that may be affected by the functionality of K^+^ channels. Cytokine transcription relies on store-operated Ca^2+^ entry (SOCE) via CRAC channels^44^. Additionally, the exocytosis of cytolytic granules is Ca^2+^-dependent^45^. A prerequisite for SOCE is a hyperpolarized membrane potential facilitated by K^+^channels^13^. KCa3.1 plays a crucial role in regulating membrane potential and SOCE^13,17^. Early evidence showed that high extracellular K^+^ concentrations depolarize NK cells and suppress cytotoxicity^46^. A recent study by Olivas-Aguirre et al.. demonstrated that blocking KCa3.1 channels in activated human NK cells suppresses SOCE^17^. Inhibition or deficiency of multiple ion channels, including CRAC channels^47^TRPM2^48^, TASK2^18^and Kv1.3^12,16,21^. In agreement with these studies, we observed that KCa3.1 inhibition suppressed cytokine, granzyme, and perforin release in NK cells, while KCa3.1 activation enhanced it. However, other groups have provided evidence of the opposite effect of KCa3.1 on cytotoxicity and showed that KCa3.1 inhibition, rather than activation, favors degranulation and cytotoxicity of NK cells in vitro^16,17^.

Koshy et al.. found that 70% of human circulating NK cells expressed KCa3.1, and KCa3.1 inhibition increased the cytotoxicity of a subpopulation of “adherent cells” (cells that have higher cytotoxicity; approximately 11% of the NK cell population)^16^. Non-adherent cells rely on Kv1.3 channels for degranulation. The nature of these adherent cells remains unclear, as they cannot be classified into specific NK cell populations such as CD16^−^CD56^dim^ or CD16^−^CD56^bright^ NK cells. Olivas-Aguirre et al. also showed that despite the inhibition of SOCE by KCa3.1, they facilitated degranulation and targeted cell killing^17^. However, they observed an initial decrease in degranulation immediately after blocking KCa3.1, which is consistent with the reduction in SOCE followed by an increase in degranulation over time. They speculated that in vitro activation could lead to excessive Ca^2+^ influx, which would harm degranulation. Other studies have shown that NK cells may require an optimal concentration of intracellular Ca^2+^ near their resting-state levels to effectively kill target cells^49^. Therefore, Olivas-Aguirre et al.. proposed that excessive intracellular Ca^2+^ triggered by activation in in vitro experiments causes immediate granule release, depleting the reserves needed for sustained killing^17^. Therefore, KCa3.1 inhibition helps to reduce Ca^2+^ and achieve effective killing.

In contrast with these reports, our findings indicate that the KCa3.1 activator NS309 enhances NK cell cytotoxicity in co-cultures with HNSCC Cal27 spheroids, whereas TRAM-34, even at concentrations sufficient to inhibit cytokine release, shows no effect. These discrepancies likely arise from differences in tumor models, such as 2D versus spheroid systems, and the higher concentrations of TRAM-34 used by others, which may lead to off-target effects.

Furthermore, our mouse data point to a distinct scenario developing in vivo. KCa3.1 activation increased NK cell tumor infiltration and cytotoxic capability, leading to reduced tumor burden. This suggests that within the immunosuppressive TME, NK cells require additional Ca²⁺ signaling to overcome suppression and sustain degranulation for effective killing. In contrast, Koshy et al.. showed an antitumor effect of KCa3.1 inhibitors in a distinct syngeneic tumor mouse model reconstituted with adherent NK cells^16^. Our translational model involving human PBMCs injected into tumor-bearing mice may account for the different outcomes observed.

Overall, our in vivo experiments demonstrated that KCa3.1 activation reduces tumor burden through immune-mediated mechanisms. Our data suggests that enhanced NK cell infiltration and cytotoxic capabilities, combined with concomitant enhancement of CD8^+^ T cells, are likely responsible for the beneficial effects of KCa3.1 activation in vivo. SKA-31 has been widely used in preclinical models and is generally well tolerated, with transient blood pressure lowering as the main off-target effect, which is manageable in the oncology setting^24,37^. Overall, these findings support SKA-31 as a suitable candidate for immunotherapy in HNSCC, although further preclinical safety evaluation remains warranted.

## Supplementary Information

Below is the link to the electronic supplementary material.

Supplementary Material 1

## Data Availability

All data needed to evaluate the conclusions of the study are presented in the paper and/or Supplementary Materials. Additional data related to this study may be requested from the authors.
