# Supplementary material for: KCa3.1 channels regulate the tumor infiltration of functionally competent NK cells in head and neck cancer
Source: Sci Rep. 2025 Oct 17;15:36360. doi: 10.1038/s41598-025-20101-x (PMC12534442; doi:10.1038/s41598-025-20101-x)
Supplement: Supplementary file 1 — Supplementary Material 1 [file 41598_2025_20101_MOESM1_ESM.docx]

**Supplemental Material**

| **Parameter** | **2 mM Ca^2+^ Ringer solution** | **0 mM Ca^2+^ Ringer solution** | ***P* value  (2 mM Ca^2+^ vs. 0 mM Ca^2+^)** |
| --- | --- | --- | --- |
| HD (n = 6) |  |  |  |
| X-COM (µm) | 8.87 ± 7.03 | -0.88 ± 2.66 | 0.437 |
| Y-COM (µm) | 41.23 ± 3.53 | 12.38 ± 3.12 | 0.001 |
| FMI^x^ | 0.03 ± 0.02 | 0.00 ± 0.02 | 0.369 |
| FMI^Y^ | 0.15 ± 0.02 | 0.07 ± 0.01 | 0.012 |
| Directness | 0.24 ± 0.01 | 0.23 ± 0.02 | 0.237 |
| Velocity (µm/sec) | 0.07 ± 0.01 | 0.04 ± 0.01 | 0.031 |
| Accumulated distance (µm) | 276.85 ± 13.34 | 178.59 ± 19.27 | 0.005 |
| Euclidean distance (µm) | 67.38 ± 3.24 | 37.33 ± 6.72 | 0.006 |

**Table S1. Role of extracellular Ca^2+^ on the chemotaxis of activated HD NK cells.** The chemotactic abilities of activated NK cells from 6 HDs towards a CXCL10 gradient were studied in Ringer solutions containing either 2 mM Ca^2+^ or 0 mM Ca^2+^. The values shown are migration parameters from the 3D chemotaxis experiment. Results are presented as mean ± SEM for all measured values. Statistical analyses were performed using either paired student’s t-test or Wilcoxon signed-rank test for non-normal distributions. X-COM and Y-COM (center of mass) are the x and y coordinates of the average point the individual migrating cells travelled by the end of the experiment: FMI (forward migration index) represents the efficiency of forward migration of the cells towards the chemokine gradient with FMI^X^ and FMI^Y^ being the FMIs in the direction of the x- and y-axes, respectively; Directness, the cells’ tendency to migrate along a straight line; Accumulated distance is the total distance traveled by the cell by the end of the experiment; Euclidean distance is the linear distance between the starting point and ending point of a cell.

| **Parameter** | **− TRAM-34** | **+ TRAM-34** | ***P* value** |
| --- | --- | --- | --- |
| HD (n = 5) |  |  |  |
| X-COM (µm) | - 8.57 ± 8.33 | 2.20 ± 9.85 | 0.492 |
| Y-COM (µm) | 92.07 ± 7.90 | - 74.12 ± 10.88 | 0.001 |
| FMI^x^ | - 0.02 ± 0.02 | - 0.01 ± 0.02 | 0.866 |
| FMI^Y^ | 0.18 ± 0.01 | - 0.15 ± 0.01 | <0.0001 |
| Directness | 0.23 ± 0.001 | 0.22 ± 0.01 | 0.125 |
| Velocity (µm/sec) | 0.17 ± 0.01 | 0.16 ± 0.02 | 0.476 |
| Accumulated distance (µm) | 517.00 ± 35.12 | 480.95 ± 44.08 | 0.469 |
| Euclidean distance (µm) | 117.42 ± 7.75 | 104.89 ± 11.73 | 0.316 |

**Table S2. Effect of TRAM-34 on the chemotaxis of activated NK cells from healthy donors.** Activated NK cells from HDs (n=5) were exposed to a gradient of either CXCL10 (-TRAM-34) or CXCL10 and 500 nM TRAM-34 (+TRAM-34). The values shown are migration parameters from the 3D chemotaxis experiment. Results are presented as mean ± SEM for all measured values. Statistical analyses were measured using paired student’s t-test. X-COM and Y-COM (center of mass) are the x and y coordinates of the average point the individual migrating cells travelled by the end of the experiment: FMI (forward migration index) represents the efficiency of forward migration of the cells towards the chemokine gradient with FMI^X^ and FMI^Y^ being the FMIs in the direction of the x- and y-axes, respectively; Directness, the cells’ tendency to migrate along a straight line; Accumulated distance is the total distance traveled by the cell by the end of the experiment; Euclidean distance is the linear distance between the starting point and ending point of a cell.

| **Parameter** | **HD (n=5)** | **HNSCC (n=5)** | ***P* value** |
| --- | --- | --- | --- |
|  |  |  |  |
| X-COM (µm) | - 8.08 ± 8.55 | -14.24 ± 7.38 | 0.624 |
| Y-COM (µm) | 92.34 ± 7.81 | 67.72 ± 6.96 | 0.049 |
| FMI^x^ | - 0.02 ± 0.02 | -0.03 ± 0.01 | 0.717 |
| FMI^Y^ | 0.18 ± 0.012 | 0.17 ± 0.02 | 0.702 |
| Directness | 0.23 ± 0.006 | 0.26 ± 0.02 | 0.351 |
| Velocity (µm/sec) | 0.17 ± 0.017 | 0.16 ± 0.03 | 0.802 |
| Accumulated distance (µm) | 517.00 ± 35.17 | 425.69 ± 73.54 | 0.295 |
| Euclidean distance (µm) | 117.42 ± 7.75 | 102.78 ± 10.84 | 0.304 |

**Table S3. Comparison of the chemotactic parameters between HD and HNSCC NK cells.** Activated NK cells from HD and HNSCC were exposed to a gradient of CXCL10 (n= 5 individuals for both groups). The HD group corresponds to the control (-TRAM-34) group from Table S2. The values shown are migration parameters from the 3D chemotaxis experiment. Results are presented as mean ± SEM for all measured values. Statistical analyses were measured using unpaired student’s t-test. X-COM and Y-COM (center of mass) are the x and y coordinates of the average point the individual migrating cells travelled by the end of the experiment: FMI (forward migration index) represents the efficiency of forward migration of the cells towards the chemokine gradient with FMI^X^ and FMI^Y^ being the FMIs in the direction of the x- and y-axes, respectively; Directness, the cells’ tendency to migrate along a straight line; Accumulated distance is the total distance traveled by the cell by the end of the experiment; Euclidean distance is the linear distance between the starting point and ending point of a cell.

| **Parameter** | **− TRAM-34** | **+ TRAM-34** | ***P* value** |
| --- | --- | --- | --- |
| HNSCC (n = 5) |  |  |  |
| X-COM (µm) | -14.24 ± 7.38 | 8.81 ± 11.10 | 0.127 |
| Y-COM (µm) | 67.72 ± 6.96 | - 39.02 ± 11.53 | 0.003 |
| FMI^x^ | -0.03 ± 0.01 | 0.02 ± 0.03 | 0.138 |
| FMI^Y^ | 0.17 ± 0.02 | - 0.13 ± 0.03 | 0.000 |
| Directness | 0.26 ± 0.02 | 0.24 ± 0.03 | 0.684 |
| Velocity (µm/sec) | 0.16 ± 0.03 | 0.14 ± 0.03 | 0.103 |
| Accumulated distance (µm) | 425.69 ± 73.54 | 346.48 ± 75.10 | 0.087 |
| Euclidean distance (µm) | 102.78 ± 10.84 | 74.36 ± 13.61 | 0.005 |

**Table S4.** **Effect of TRAM-34 on the chemotaxis of activated NK cells from HNSCC patients.** Activated NK cells from HNSCC patients (n = 5) were exposed to a gradient of either CXCL10 (-TRAM-34) or CXCL10 and 500 nM TRAM-34 (+TRAM-34). The values shown are migration parameters from the 3D chemotaxis experiment. Results are presented as mean ± SEM for all measured values. Statistical analyses were measured using paired student’s t-test. X-COM and Y-COM (center of mass) are the x and y coordinates of the average point the individual migrating cells travelled by the end of the experiment: FMI (forward migration index) represents the efficiency of forward migration of the cells towards the chemokine gradient with FMI^X^ and FMI^Y^ being the FMIs in the direction of the x- and y-axes, respectively; Directness, the cells’ tendency to migrate along a straight line; Accumulated distance is the total distance traveled by the cell by the end of the experiment; Euclidean distance is the linear distance between the starting point and ending point of a cell.

| **Parameter** | **− ADO** | **+ ADO** | **+ADO +NS309** | ***P* value (- ADO versus + ADO)** | ***P* value (+ ADO versus + ADO +NS309)** |
| --- | --- | --- | --- | --- | --- |
| HNSCC (n = 3) |  |  |  |  |  |
| X-COM (µm) | - 0.93 ± 11.66 | - 5.48 ± 5.80 | 3.27 ± 6.82 | 0.582 | 0.490 |
| Y-COM (µm) | 55.22 ± 3.13 | - 14.29 ± 3.15 | 71.04 ± 5.28 | 0.000 | 0.003 |
| FMI^x^ | 0.01 ± 0.04 | - 0.01 ± 0.01 | 0.01 ± 0.02 | 0.313 | 0.663 |
| FMI^Y^ | 0.19 ± 0.01 | - 0.08 ± 0.01 | 0.23 ± 0.02 | 0.250 | 0.006 |
| Directness | 0.27 ± 0.01 | 0.28 ± 0.04 | 0.34 ± 0.01 | 0.835 | 0.415 |
| Velocity (µm/sec) | 0.06 ± 0.00 | 0.04 ± 0.008 | 0.06 ± 0.00 | 0.127 | 0.133 |
| Accumulated distance (µm) | 297.54 ± 20.34 | 197.88 ± 28.27 | 307.27 ± 11.21 | 0.025 | 0.025 |
| Euclidean distance (µm) | 79.62 ± 5.75 | 51.96 ± 3.01 | 100.72 ± 7.62 | 0.081 | 0.044 |

**Table S5. Effect of ADO and KCa3.1 channel activation on the chemotaxis of activated HNSCC NK cells.** Activated NK cells from HNSCC patients were treated to either a gradient of either CXCL10 (-ADO), CXCL10 and 10 µM ADO (+ADO) or HNSCC NK cells were preincubated with 1 μM NS309 and exposed to a CXCL10 and ADO gradient (+ADO+NS309) (n= 3 HNSCC patients). The values shown are migration parameters from the 3D chemotaxis experiment. Results are presented as mean ± SEM for all measured values. Statistical analyses were measured using paired student’s t-test. X-COM and Y-COM (center of mass) are the x and y coordinates of the average point the individual migrating cells travelled by the end of the experiment: FMI (forward migration index) represents the efficiency of forward migration of the cells towards the chemokine gradient with FMI^X^ and FMI^Y^ being the FMIs in the direction of the x- and y-axes, respectively; Directness, the cells’ tendency to migrate along a straight line; Accumulated distance is the total distance traveled by the cell by the end of the experiment; Euclidean distance is the linear distance between the starting point and ending point of a cell.

**
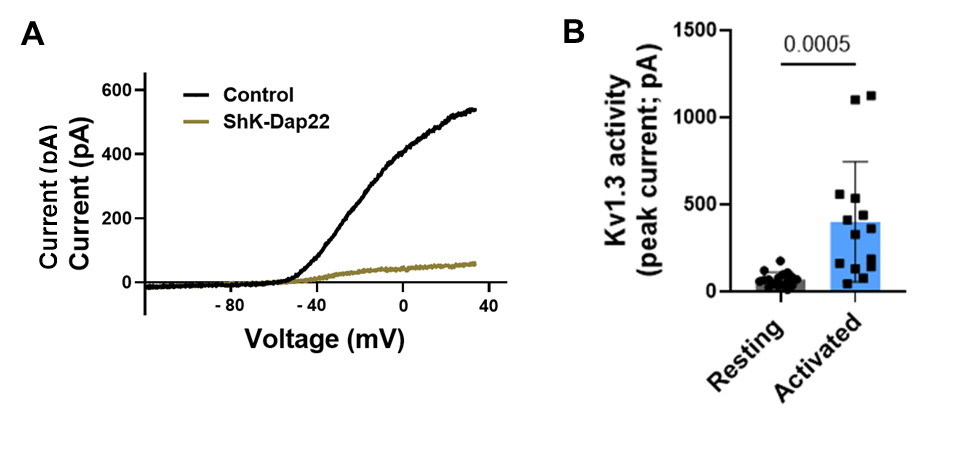
**

**Fig. S1. Increase in functional Kv1.3 channels in NK cells with activation.** (**A**) Representative Kv1.3 current traces before (control) and after treatment with the selective Kv1.3 blocker ShK-Dap22 (10 nM). (**B**) Average Kv1.3 peak current (measured at ±50 mV) as an indicator of channel activity from 17 resting cells and 14 activated NK cells isolated from 4 HDs. Data were analyzed by unpaired student’s t-test and are reported as mean ± SD. Each symbol in the graph corresponds to measurements obtained from a single cell.


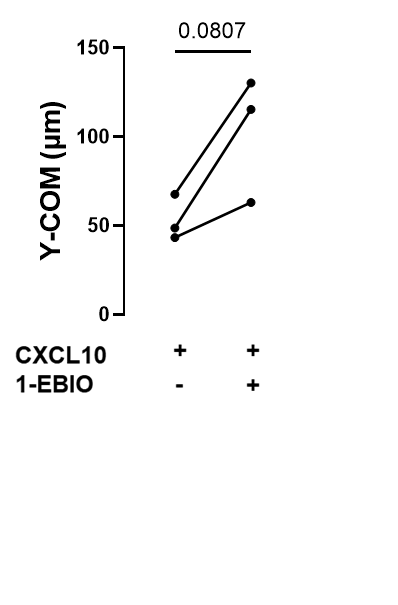


**Fig. S2. Increase in chemotaxis of primary NK cells in the presence of the KCa3.1 activator 1-EBIO.** Y-COM values of activated NK cells migrating towards a CXCL10 gradient with and without pretreatment with 1-EBIO (20 µM) for 1 h. The experiment was conducted with samples from three HDs. Data were analyzed by paired t-test.


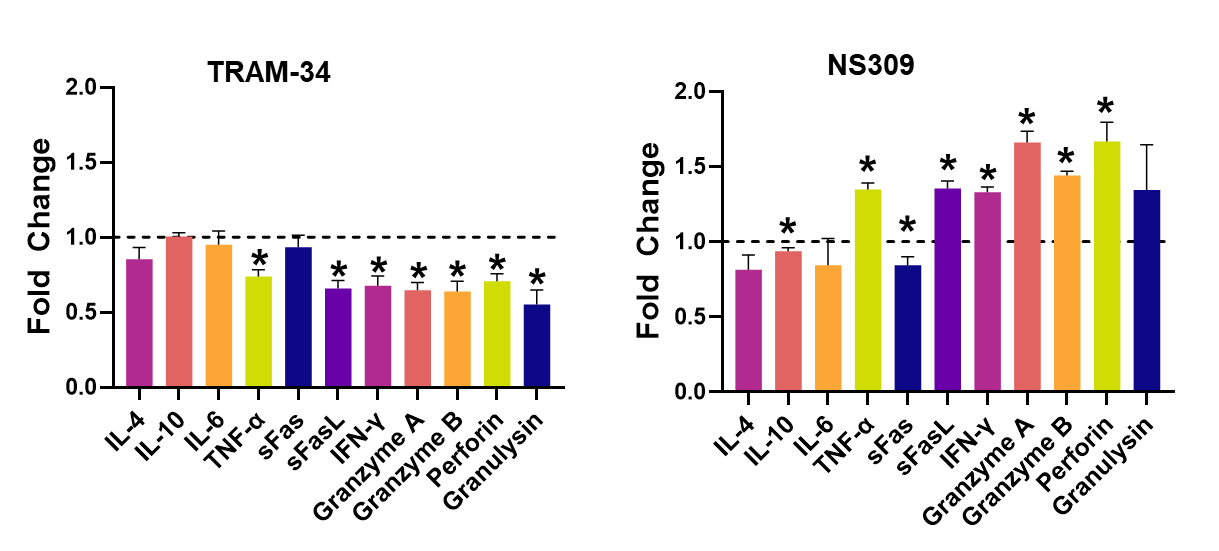


**Fig. S3.** **KCa3.1 modulates the release of cytokines and cytotoxicity markers by NK cells activated with IL-12 and IL-18.** Multiplex cytokine release assay showing fold change in the abundance of individual proteins (cytokine and cytotoxicity markers) in primary NK cells that were stimulated with IL-12 and IL-18 for 24 h and treated with either 500 nM TRAM-34 (left) or 1 µM NS309 (right). Vehicle treated cells were used as controls, and the abundance of the individual analyte represented in the bars are relative to those in controls (dotted line). Data were measured in n = 5 HDs (the same donors were used for both treatments). Statistical analysis was performed by Wilcoxon signed-rank test. The bars represent the mean ± SD. * p <0.05.


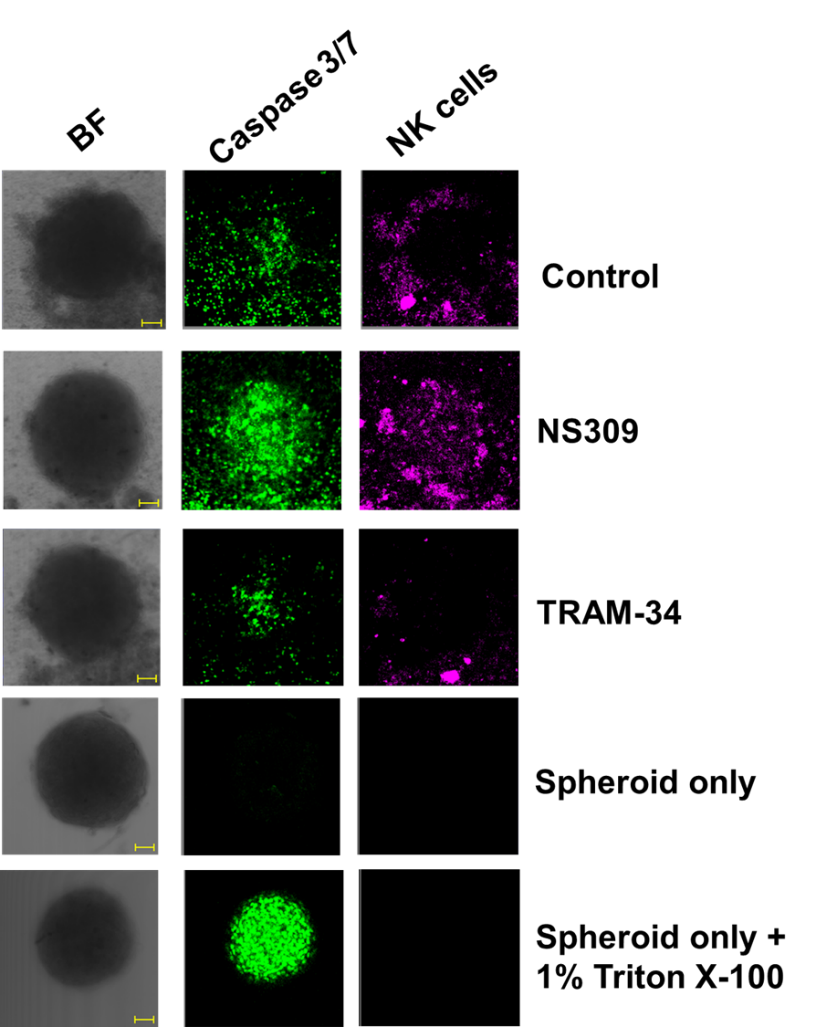


**Fig. S4. KCa3.1 affects the ability of activated NK cells to kill HNSCC cells.** Representative confocal images illustrating the killing of Cal27 spheroids co-cultured with activated NK cells, which were loaded with Cell Tracker Deep Red Dye (magenta), at an effector-to-target ratio of 10:1 for 3 h. NK cells were activated without any treatment (control) or in the presence of 500 nM TRAM-34 (KCa3.1 inhibitor) or 1 μM NS309 (KCa3.1 activator). Cell death was indicated by the abundance of caspase 3/7 (green). Spheroids without NK cells served as negative controls, whereas spheroids (without NK cells) treated with 1% Triton X-100 were used as positive controls for cell death. Scale bars, 50 μm.

**
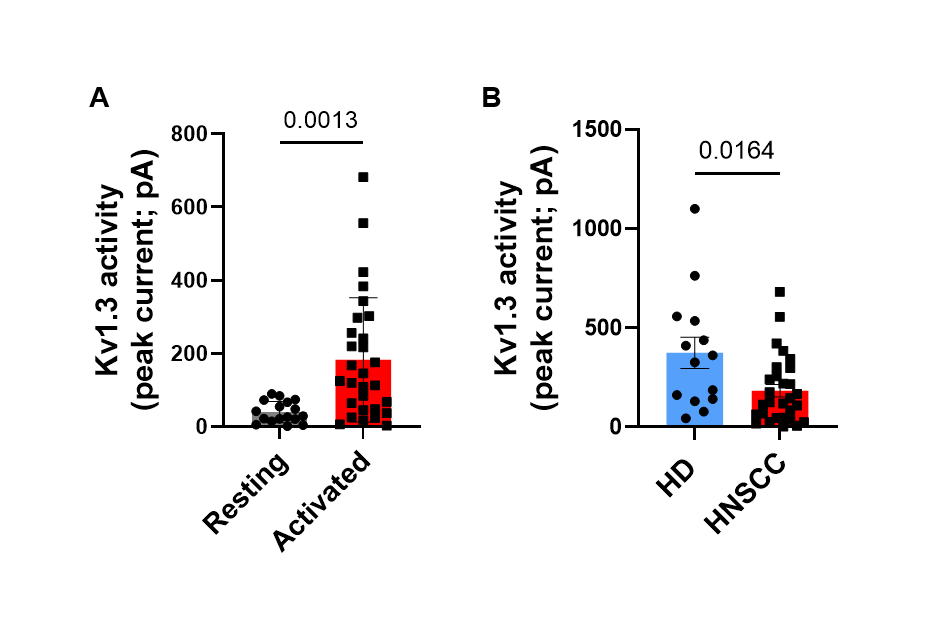
**

**Fig. S5. Functional Kv1.3 channels are reduced in activated NK cells from HNSCC patients as compared to healthy donors.** (**A**) Kv1.3 peak currents measured in resting and activated NK cells from HNSCC patients (n=17 resting and 29 activated cells from 8 HNSCC patients). (**B**) Kv1.3 activity measured in activated NK cells from healthy donors (HD) and HNSCC patients (n=14 cells measured in 4 HDs and n=29 cells measured in 8 HNSCC patients). Statistical analyses were performed by unpaired student’s t-test for (**A**) and Mann-Whitney test for (**B**). Bars represent mean ± SD. Each symbol represents an individual cell.


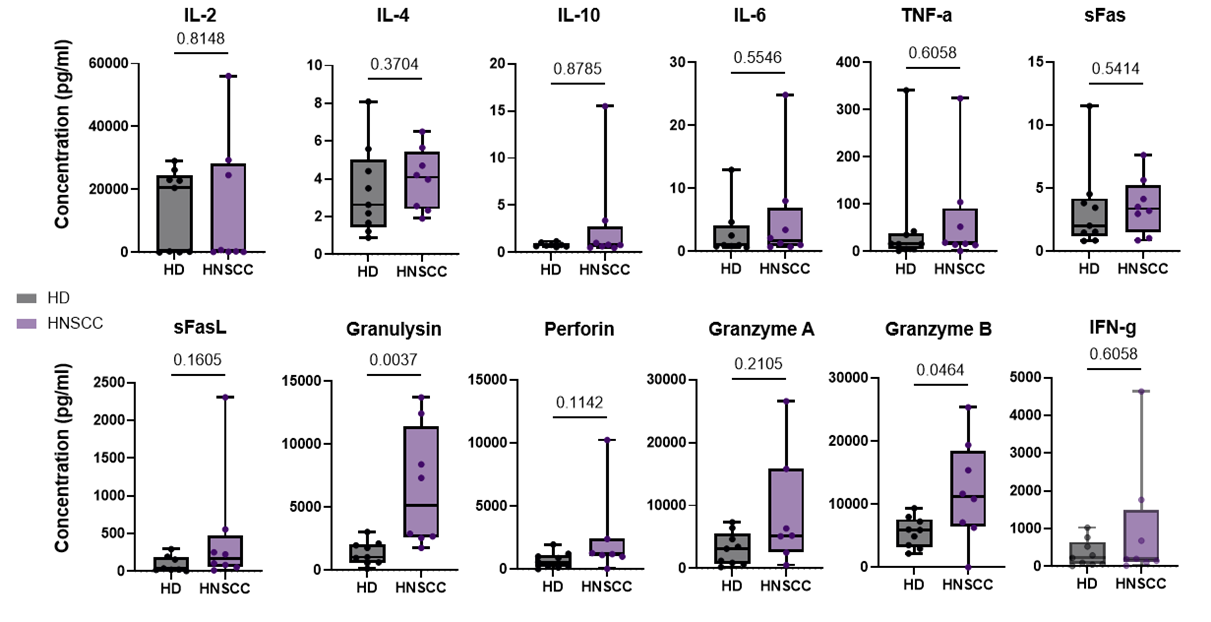


**Fig. S6. Similar cytokine and cytotoxicity marker release profiles of HD and HNSCC NK cells.** Abundance (pg/ml) of cytokines released by NK cells from HDs (n= 8-9) and HNSCCs (n= 8) stimulated with IL-2 and IL-15 for 48 h. Data are visualized as box and whisker plots. The data are reported as the median (horizontal line), first (top box) and third quartiles (bottom box), and each symbol represents an individual HD or HNSCC patient. Statistical analyses were performed by Mann-Whitney test.
